# The steroid hormone estriol (E_3_) regulates epigenetic programming of fetal mouse brain and reproductive tract

**DOI:** 10.1186/s12915-022-01293-4

**Published:** 2022-05-02

**Authors:** Yuping Zhou, Baoxia Gu, Geraldine Brichant, Jay Prakash Singh, Huan Yang, Hao Chang, Yanding Zhao, Chao Cheng, Zhong-Wu Liu, Myles H. Alderman, Lingeng Lu, Xiaoyong Yang, Xiao-Bing Gao, Hugh S. Taylor

**Affiliations:** 1 Department of Obstetrics, Gynecology and Reproductive Sciences, Yale School of Medicine, Yale University, 333 Cedar Street, New Haven, CT 06520 USA; 2 Present Address: Reproductive Medicine Center of Henan Provincial People’s Hospital, People’s Hospital of Zhengzhou University, Zhengzhou, Henan People’s Republic of China 450003; 3 Department of Comparative Medicine and of Cellular and Molecular Physiology, Yale University, New Haven, CT 06520 USA; 4 Department of Genetics, Yale University, New Haven, CT 06520 USA; 5 Department of Medicine, Dan L Duncan Comprehensive Cancer Center, Baylor College of Medicine, Houston, TX 77030 USA; 6 Present Address: Yale Stem Cell Center, Yale University, New Haven, CT 06520 USA; 7 Department of Chronic Disease Epidemiology, Yale University, New Haven, CT 06520 USA

**Keywords:** Estriol, E_3_, Epigenetics, DNA methylation, Behavior, Reproduction, Pregnancy, Developmental programming

## Abstract

**Background:**

Estriol (E_3_) is a steroid hormone formed only during pregnancy in primates including humans. Although E_3_ is synthesized at large amounts through a complex pathway involving the fetus and placenta, it is not required for the maintenance of pregnancy and has classically been considered virtually inactive due to associated very weak canonical estrogen signaling. However, estrogen exposure during pregnancy may have an effect on organs both within and outside the reproductive system, and compounds with binding affinity for estrogen receptors weaker than E_3_ have been found to impact reproductive organs and the brain. Here, we explore potential effects of E_3_ on fetal development using mouse as a model system.

**Results:**

We administered E_3_ to pregnant mice, exposing the fetus to E_3_. Adult females exposed to E_3_ in utero (E_3_-mice) had increased fertility and superior pregnancy outcomes. Female and male E_3_-mice showed decreased anxiety and increased exploratory behavior. The expression levels and DNA methylation patterns of multiple genes in the uteri and brains of E_3_-mice were distinct from controls. E_3_ promoted complexing of estrogen receptors with several DNA/histone modifiers and their binding to target genes. E_3_ functions by driving epigenetic change, mediated through epigenetic modifier interactions with estrogen receptors rather than through canonical nuclear transcriptional activation.

**Conclusions:**

We identify an unexpected functional role for E_3_ in fetal reproductive system and brain. We further identify a novel mechanism of estrogen action, through recruitment of epigenetic modifiers to estrogen receptors and their target genes, which is not correlated with the traditional view of estrogen potency.

**Supplementary Information:**

The online version contains supplementary material available at 10.1186/s12915-022-01293-4.

## Background

Estrogens, synthesized predominantly in the ovary, are steroid hormones essential for development of female sex characteristics and reproduction. There are three common mammalian estrogens: estrone (E_1_), estradiol (E_2_), estriol (E_3_). E_2_ is the primary circulating and most potent estrogen produced by the ovaries in premenopausal women. E_3_, a 16α-hydroxylated metabolite of E_2_ and E_1_, is present at very low levels in non-pregnant women and is considered a much less potent estrogen than E_2_ [1, 2]. Thus, E_3_ likely contributes little to the overall estrogenic activity in non-pregnant, premenopausal women [3]. However, during pregnancy E_3_ is produced in prodigious quantities by a unique and complex pathway that involves three organs of the feto-placental unit: the adrenals, liver, and placenta. In humans, E_3_ accounts for more than 90% of total estrogens circulating in pregnancy [2, 4]. This feto-placental driven synthesis begins with the △^5^-3β-hydroxy-C_19_-steroid dehydroisoandrosterone sulfate (DS), synthesized and secreted by the fetal adrenals, which undergoes 16α-hydroxylation by an unusual hepatic enzyme, a 16α-hydroxylase capable of acting on a non-aromatic and even more unusually, an ionic substrate [5]. This product, 16α-hydroxy-dehydroisoandrosterone sulfate, is then converted into E_3_ in the placenta through processing by several different enzymes, most importantly, sulfatase, several dehydrogenases, and most notably, aromatase [5].

While it has been known for decades that in humans and several other primates [5], E_3_ is produced in much greater quantities than other estrogens by this pregnancy-specific pathway, its physiological role, if any, remains unknown. In humans, the productions of estrogens, including E_3_, are not required for the maintenance of pregnancy [6]. However, it is now recognized that estrogen exposure during gestation can have profound effects on organs both within the reproductive system and outside the reproductive system including the brain, breast, and cardiovascular system. Indeed, estrogens are known to have important effects on a wide variety of social behaviors, such as aggression, self-recognition, anxiety, communication, and learning [7]. More recently, it has been found that many so-called “weak estrogens” can have actions that would not be predicted on the basis of their binding affinity to the ER or their effect on the vagina and uterus. Of note, several compounds with weaker binding affinity for ERs than E_3_ have an important effect on reproductive organs and the developing brain during pregnancy [8]. Thus, this has led us to suspect that E_3_ might have a similar role. Estrogens can cross both the placenta and the blood brain barrier [9], and E_3_, unlike E_2_, binds only poorly to sex hormone-binding globulin and therefore can traverse the blood brain barrier more readily than the “more potent” hormone [10]. Whether E_3_ influences fetal brain development and subsequent adult offspring behavior is unknown and the subject of this study in mice. However, rodents do not normally produce E_3_ during pregnancy. Thus, we applied a mini-osmotic pump to continuously release E_3_ in pregnant mice, mimicking E_3_ production during human pregnancy. Our present study has found that during pregnancy E_3_ exposure subsequently shapes adult offspring reproduction and behavior by epigenetically programming the fetus. E_3_ alters the expression levels and DNA methylation patterns of many genes in the uterus and brain of exposed animals. Mechanistically, E_3_ affects complexing of ERs with several DNA/histone modifiers, and the binding of these complexes to target genes. Rather than a weak estrogen as defined by its function as a canonical transcriptional activator, E3 could be a potent epigenetic modulator and influence developmental programming of the fetus.

## Results

### Fetal E_3_ exposure improves reproductive success

**Fig. 1:**
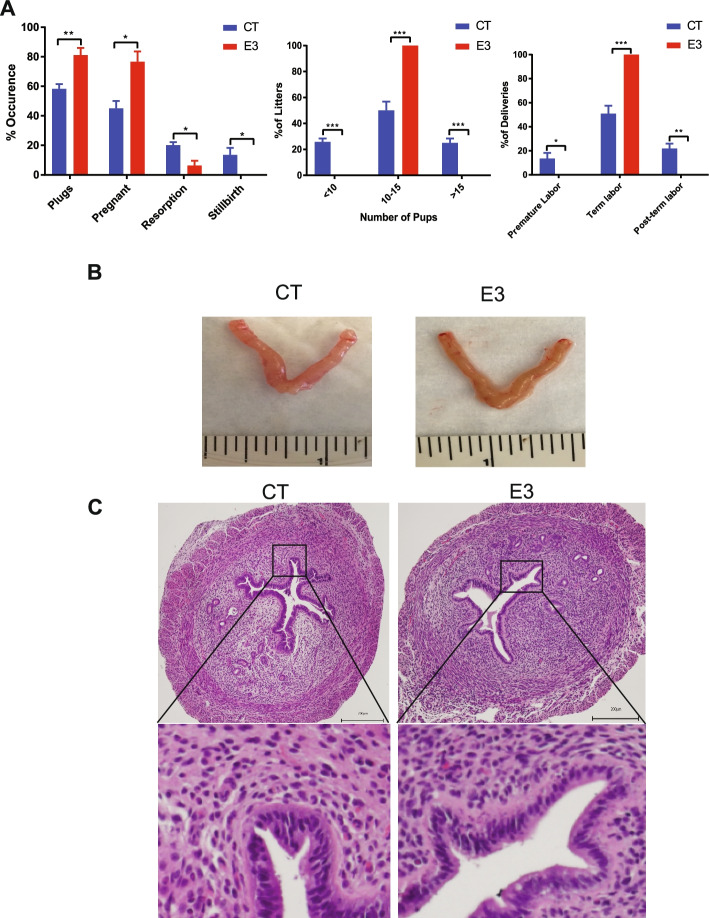
Fetal E_3_ exposure improves reproduction in female offspring. Eight-week-old pregnant female CD-1 mice were treated with vehicle DMSO (CT) or E_3_ (E3). At 8 weeks after birth, the female offspring were bred to untreated male CD-1 mice. **A** The percentage of pregnancy occurrence (vaginal plug, pregnancy, resorption, and stillbirth), litter size (number of pups), and percentage of deliveries (premature labor, term labor, and post-term labor). Error bars: mean + SEM. *, *p* < 0.05; **, *p* < 0.01; ***, *p* < 0.001 by Student *t* test, *N* = 55 CT, 40 E_3_-treated female offspring. **B** The uterine morphology of control (CT) and E_3_ female offspring that had received treatment as a fetus. **C** Representative micrographs of hematoxylin and eosin staining of the uteri. The images are representative of 10 mice

Estrogens are critical for the development of female reproductive organs and normal adult physiologic function. While the role of E_2_ is well defined, there is no known physiological role for E_3_. To determine the effect of fetal E_3_ exposure on subsequent reproductive success, we treated pregnant mice with E_3_ or vehicle (CT) and then examined the reproductive ability of the female offspring (designated E3 and CT). Vaginal plug, rate of pregnancy, resorption, premature labor, stillbirth, and litter size were recorded. The E3 mice were more likely to mate than CT mice, with 81% (32 out of 40 mice) of E3 versus 58% (32 out of 55 mice) of CT displaying plugs (*p* = 0.009). The E_3_ mice also had a higher chance of pregnancy success than CT mice after mating (76% versus 45%). Similarly, the E3 mice had a lower rate of resorption than CT (6% versus 20%) and no stillbirths (0% versus 14%). The E3 mice produced a stable litter size of 10–15 pups per litter, while 25% of CT gave birth to either <10 pups or >15 pups per litter. Some CT mice had premature labor (14% delivery on E18) and post-term labor (22% delivery on E22), while all E3 mice had term labor (100% delivery on E20)) (Fig. 1A). No differences were detected in the body weights of the pups between the two groups. The uteri appeared grossly and histologically similar (Fig. 1B, C). These results suggest that in utero exposure to E_3_ can improve the reproductive success of female offspring.

### Prenatal E_3_ exposure persistently alters adult uterine gene expression and methylation

**Fig. 2:**
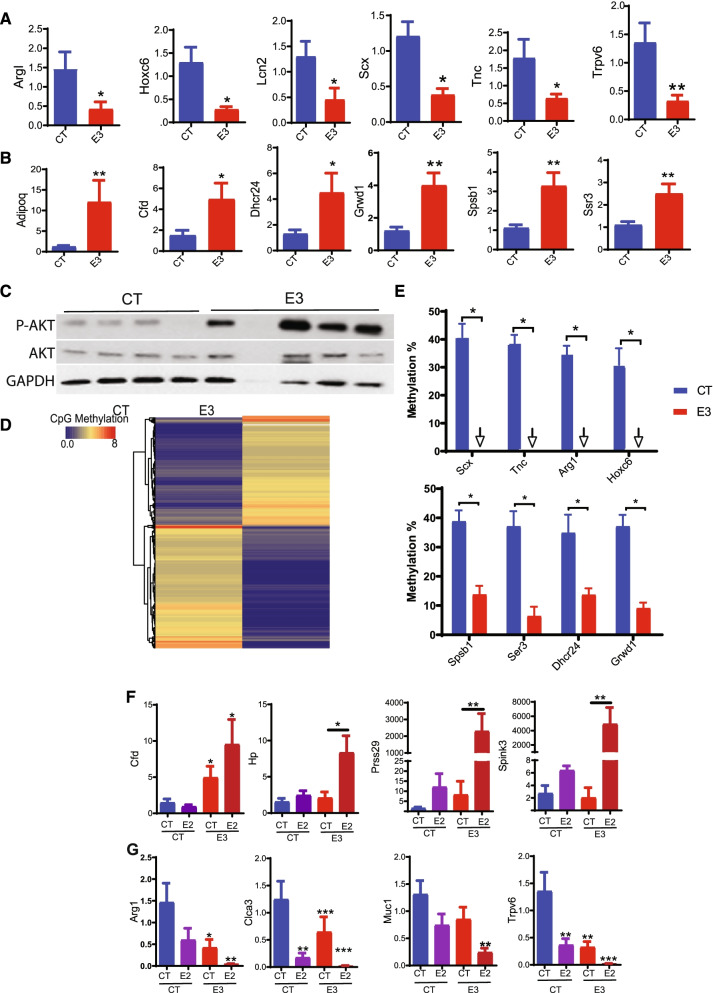
Prenatal exposure to E_3_ alters global gene expression, DNA methylation, pAKT protein levels, and E_2_ responsiveness in female offspring uteri. Eight-week-old pregnant female CD-1 mice were treated with vehicle DMSO (CT) or E_3_. At 8 weeks after birth, the female offspring uteri were analyzed. **A, B** Quantification of selected gene expression in uteri by quantitative RT-PCR. **A** The representative genes with decreased expression after fetal E_3_ treatment. **B** The representative genes with increased expression after fetal E_3_ treatment. **C** Immunoblots of total and phosphorylated (p) Akt in the uteri of vehicle (CT) and E_3_-treated mice (E_3_). **D** Heat maps showing the top 3000 differential CpG sites with altered methylation in E_3-_treated compared to control uterus. **E** Representative demethylated genes with altered expression from **A** and **B**. Error bars: mean + SEM. *, *p* < 0.05; **, *p* < 0.01; ***, *p* < 0.001 by Student *t* test, *n* = 8 mice/group. Programming of estrogen response: **F, G** Pregnant CD-1 mice were prenatally exposed to E_3_ (E3) or vehicle (CT). Six weeks after birth, the female offspring were ovariectomized; after 2 weeks they were transiently (6hrs) treated with either E_2_ or vehicle control as adults (designated CT-CT, CT-E2, E3-CT, and E3-E2, where the first term prior to the dash signifies prenatal exposure and the second term after the dash is the transient adult treatment). **F, G** Quantification of selected gene expression by quantitative RT-PCR. **F** The representative genes demonstrating an exaggerated estrogen response as adults after prenatal E_3_ exposure. **G** The genes where enhanced repression in response to E_2_ exposure was programmed by fetal E_3_ exposure. Error bars: mean + SEM. *, *p* < 0.05; **, *p* < 0.01; ***, *p* < 0.001 by Student *t* test, *n* = 12 mice/group. Uncropped blots available in Additional file 3: Fig. S7

Previous studies in this laboratory demonstrated that xenoestrogens such as Bisphenol A and diethylstilbestrol could alter developmental programming through epigenetic modification [11, 12]. Thus, we postulated that E_3_ might be a natural epigenetic modifier capable of programming significant changes in gene transcription in the adult. Microarray analysis was performed using cDNA pooled from 6 mice per experimental group. Our results show that prenatal exposure to E_3_ had a significant impact on the uterine global gene expression profile. As adults, a total of 610 genes demonstrated a permanently altered expression pattern (274 upregulated and 336 downregulated) in the in utero E_3_-treated offspring compared to vehicle-treated controls (Additional file 2: Table S2). Among all the altered genes, 421 genes could be classified based on biological process or molecular function (Additional file 1: Fig. S1A). Notably, there was a significant enrichment in cancer-related genes (66 genes), consistent with the known role of xenoestrogens in tumor development. The second largest category was cellular growth and proliferation (58 genes), followed by 57 genes involved in cell death. Representative genes putatively associated with reproduction and carcinogenesis were selectively validated by RT-qPCR. As shown in Fig. 2A, the mRNA expression of *Arg1* (arginase type I), *Hoxc6* (homeobox C6), *Lcn2* (lipocalin-2), *Scx* (basic helix-loop-helix transcription factor scleraxis), *Tnc* (tenascin C), and *Trpv6* (transient receptor potential cation channel) were reduced by more than 3-fold. While the transcripts of *Adipoq* (adiponectin), *Cfd* (complement factor D), *Dhcr24* (24-dehydrocholesterol reductase), *Grwd1* (Glutamate-Rich WD Repeat Containing 1), *Spsb1* (splA/ryanodine receptor domain and SOCS box containing 1), and *Ssr3* (signal sequence receptor, gamma) were upregulated by over 2-fold in the in utero E_3_-treated offspring (E3) compared to vehicle-treated controls (CT) (Fig. 2B).

Some of the identified genes could be assigned to a specific cellular pathway/signaling network (Additional file 1: Fig. S1B). For example, 17 genes, 6 of which were downregulated and 11 upregulated, were directly or indirectly involved in the protein kinase B (Akt or PKB)/caspase / 26S proteasome signaling. Indeed, Akt phosphorylation was enhanced significantly in the in utero E_3_-treated offspring (E3) compared to vehicle-treated controls (CT) (Fig. 2C).

DNA methylation is a crucial part of normal organismal development and cellular differentiation in higher organisms by stably altering gene expression. We hypothesized that transient fetal exposure to E_3_ would epigenetically modify numerous genes, leading to a permanent change in expression pattern that persisted into adulthood with an effect on reproductive function. To this end, we performed a DNA methylation array, which accurately identifies methylated DNA loci including CpG islands. We found significant differential methylation in the uteri of E_3_-treated mice (Fig. 2D). We identified 2233 genes that were significantly hypomethylated in the E_3_-treated group (E3) compared to non-treated group CT (Additional file 2: Table S3A). Most of the altered genes were involved in body development, including the reproductive and hematological systems (Additional file 1: Fig. S2A). The enriched molecular function categories included enzymes (100 genes) and transcription factors (94 genes) (Additional file 1: Fig. S2B). Pathway analyses of the hypomethylated genes revealed that 20 genes were potentially associated with the HOXA5-PKD1-CREB-CTNNB1 signaling network involving signal transduction, cell-to-cell communication, and transcription regulation (Additional file 1: Fig. S2C). Thirty-three genes were in the HNF1 (hepatocyte nuclear factor 1 homeobox A) -RORA (nuclear hormone receptor) network (Additional file 1: Fig. S2D).

We next asked if E_3_-induced gene demethylation correlated with altered gene expression. Indeed, 4 (S*cx*, *Tnc*, *Arg1*, and *Hoxc6*) out of 6 downregulated genes, and 4 (*Spsb1*, *Ssr3*, *Dhcr24*, and *Grwd1*) out of 6 upregulated genes confirmed above (Fig. 2E) were also specifically and directly hypomethylated by in utero E_3_ treatment (E3) compared to vehicle controls (CT).

We also identified 2557 genes that were hypermethylated (Additional file 2: Table S3B), 91 of which were associated with nervous system development (Additional file 1: Fig. S3A). The enriched molecular function categories primarily included G-protein coupled receptors (GPCR) (97 genes), enzymes (45 genes), and transcription factors (46 genes) (Additional file 1: Fig. S3B). Pathway analyses of the hypermethylated genes revealed 3 major networks, 12 genes in the INS1-CEBPB signaling network (endocrine system development and function) (Additional file 1: Fig. S3C), and 14 genes in the ERK1-AKT network (cellular and connective tissue development and function) (Additional file 1: Fig. S3D). **.**

### In utero exposure to E_3_ alters the E_2_ responsiveness as adults

We have previously shown that in utero exposure to xenoestrogens specifically programs estrogen response in the exposed animals as adults [11–14]. Here we treated pregnant mice with E_3_ or vehicle (CT) and then examined the gene expression profiles of the offspring that had received subsequent adult treatment with either E_2_ (CT-E2, E3-E2) or vehicle control (CT-CT, E3-CT). We used the first of the two terms to designate fetal exposure and the second term to designate subsequent adult treatment (i.e., in utero exposure- adult treatment). An additional set of genes showed altered response only after E_2_ stimulation (E3-E2); expression of 114 genes was upregulated in response to E_2_ stimulation in E_3_-treated (E3-E2) offspring (Additional file 2: Table S4) compared to CT-E2. A group of genes including *Cfd* (adipsin), *Hp* (haptoglobine), *Prss29* (protease, serine 29), and *Spink3* (serine protease inhibitor, Kazal-type 1) were upregulated dramatically only in E3-E2 group compared to CT-CT, CT-E2, or E3-CT groups. *Cfd* (adipsin) was upregulated significantly in the E3-E2 group compared to CT-CT or CT-E2, but not to the E3-CT group (Fig. 2F). These genes were normally either non-responsive to estrogen treatment or responded only modestly, suggesting that E_3_ programming can lead to the development of a profound E_2_ responsiveness.

We also examined genes that showed decreased responsiveness to acute E_2_ treatment as adults after E_3_ exposure. The mRNA levels of genes including *Arg1* (arginase 1), *Clca3* (chloride channel accessory 3), *Muc1* (mucin 1), and *Trpv6* (transient receptor potential cation channel) were reduced modestly (<6 fold) in E3-CT group compared to the CT-CT or CT-E2 group, and more dramatically decreased (>6 fold) in E3-E2 compared to CT-E2 (Fig. 2G). Taken together, these results indicate that in utero exposure of E_3_ during pregnancy permanently alters the E_2_ responsiveness as adults.

### Prenatal E_3_ exposure increases the expression of epigenetic (DNA/histone) modifiers

**Fig. 3:**
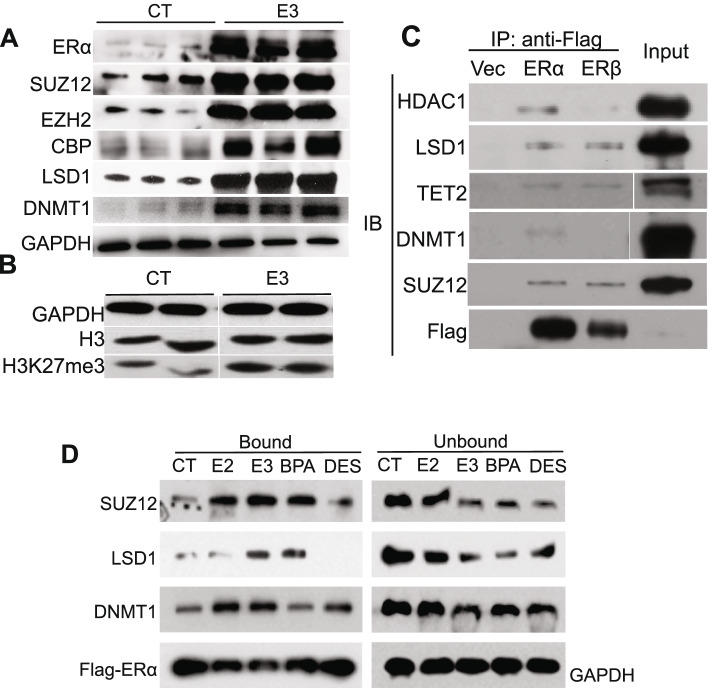
Prenatal exposure of E_3_ enhances expression of epigenetic modifiers in female offspring and enhances ER binding to epigenetic modifiers. **A** Representative immunoblots of epigenetic modifying proteins from the uteri of prenatally vehicle- (CT, *n* = 3 mice) and E_3_-treated mice (E3, *n* = 3 mice). **B** Increased histone methylation in prenatally E_3_-treated mice (*n* = 2 mice) compared to those treated with vehicle control (*n* = 2 mice). **C** Co-immunoprecipitation (IP) of ERα/β with DNA/histone modifiers from Ishkawa cells expressing Flag (Vec), Flag-ERα, or Flag-ERβ respectively, using an anti-Flag antibody followed by immunoblotting (IB) using the indicated antibodies. **D** Co-IP of Flag-ERα with DNA/histone modifiers from Ishkawa cells treated with DMSO (CT), E_2_ (E2), E_3_ (E3), bisphenol A (BPA), or diethylstilbestrol (DES). The immunoblots show the proteins bound to Flag-ERα and are representative of 3 independent experiments. Uncropped blots available in Additional file 3: Fig. S7

We have noted that in utero E_3_ treatment permanently altered global gene expression and the DNA methylation profile of the F1 generation. ER signaling has been previously demonstrated to induce DNA methyl transferase expression [15] [16]. We then asked if E_3_ also regulates expression of DNA and histone modifiers. We examined the expression of several DNA and histone modifiers including DNA methyl transferase I (DNMT1) and enhancer of Zeste homolog 2 (EZH2, histone lysine N-methyltransferase) histone 3 lysine 27 methyl transferase (H3K27me), histone lysine-specific demethylase (LSD1), suppressor of Zeste 12 Protein (SUZ12), and CREB-binding protein (CBP, histone acetyltransferase). The protein levels of SUZ12, EZH2, and CBP were higher in all mice exposed to E_3_ than those of controls; similarly, LSD1 and DNMT1 protein levels were also moderately increased in the E_3_-treated group **(**Fig. 3A). To demonstrate that the increased expression led to altered histone methylation, we examined H3K27 methylation in the E_3_ and control groups and noted increased H3K27me in the fetal E_3_-exposed offspring (Fig. 3B).

### Epigenetic modifiers bind ER

The effects of endocrine disruptors such as DES require ER. We also hypothesized that the differential effects of distinct estrogens on epigenetic alteration may be mediated by differential conformational changes in ER as are known to be induced by DES or several selective estrogen receptor modulators (SERMs). These changes may alter the complement of ERα/β interacting proteins. We over-expressed Flag-tagged ERα or ERβ in Ishkawa cells and co-immunoprecipitated the Flag-ER and its bound proteins using an anti-Flag antibody. Potential proteins bound by ERα or ERβ were identified by Multi-Dimensional Protein Identification Technology (MudPIT). We identified 1322/2398 proteins that directly bound to ERα/ERβ (Additional file 2: Table S5**)**. We identified several epigenetic modifiers including histone deacetylase 1(HDAC1), LSD1, methylcytosine dioxygenase 2 (TET2), DNMT1, and SUZ12. These interactions were validated by immunoblotting (Fig. 3C). We noted that Flag-ERβ demonstrated higher affinity for LSD1, TET2, and SUZ12 than Flag-ERα (when taking the amounts of Flag-ERs inputs into account), while Flag-ERα bound more HDAC1 and DNMT1 than did Flag-ERβ.

We next asked if estrogen treatment would differentially modulate the binding of ERs to these epigenetic modifiers. Flag-ERα-expressing Ishkawa cells were pretreated with E_2_, E_3_, BPA, or DES before cells were lysed for immunoprecipitation. Compared to control (CT), the E_2_, E_3_, and BPA treatments enhanced ERα binding capacity of SUZ12, LSD1, and DNMT1 to various degrees. ERα binding to SUZ12 was increased most evidently by E_2_, E_3_, and BPA; binding to LSD1 was enhanced most dramatically by E_3_ and BPA; and E_2_ and E_3_ increased ERα binding to DNMT1 most significantly. ERα binding to SUZ12 and DNMT1 remained largely unchanged and LSD1 was surprisingly reduced after treatment with diethylstilbestrol (DES), a synthetic non-steroidal estrogen (Fig. 3D). To further confirm that E_3_ activates ER binding to DNA modifiers, which together bind to the target genes, we performed a Re-ChIP assay. We identified 352 DNA sequences bound by both ERα and SUZ12 (Additional file 2: Table S6 A-B), E_3_ treatment enhanced/decreased the ERα-SUZ12 binding to 175/74 genes respectively (Additional file 1: Fig. S2E), and 1099 sequences by ERβ and SUZ12 simultaneously (Additional file 2: Table S6 C-D). E_3_ treatment enhanced/decreased the ERβ-SUZ12 binding to 456/84 genes respectively (Additional file 1: Fig. S2F).

### Fetal E_3_ exposure reduces anxiety-like behavior in adult offspring

In addition to the reproductive tract, the brain is a highly sexually dimorphic organ that is responsive to estrogen signaling. Estrogens modulate multiple neural functions, including mood, anxiety, fear, and higher-order cognitive functions by enhancing learning and memory [17, 18]. We performed a water maze test and novel object recognition (NOR) task to investigate the impact of fetal E_3_ on learning and memory behavior. There were no differences in the time to escape, passing time, time spent in the area, and path length between E_3_ and CT mice (Additional file 1: Fig. S4 A-D). There were no significant differences in the amount of time taken to explore the new object between E_3_ and CT mice (Additional file 1: Fig. S4E). Both behavioral tests demonstrate that E_3_ treatment did not affect mouse memory and recognition.

**Fig. 4:**
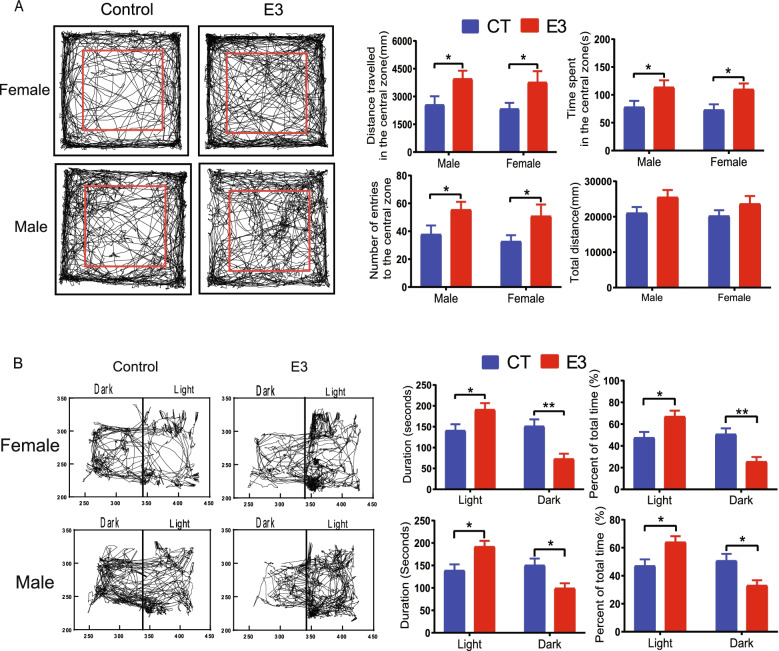
Prenatal exposure to E_3_ reduces the anxiety level of adult offspring. Eight-week-old pregnant female CD-1 mice were treated with vehicle DMSO (CT) or E_3_ (E3). At 6 months after birth, the offspring were subjected to a 20 min-open field test and light/dark transition test. **A** A representative result of the open field test. The red rectangles indicate the central area of Path tracking. The distance travelled in millimeters that a mouse travelled in the central zone. The total time in seconds that a mouse spent in the central zone. The number of entries in the central zone. The total distance in millimeters that a mouse travelled in the whole field. **B** A representative result of the light/dark transition test. Path tracking in the light/dark compartment; Duration (seconds): the time spent in the light/dark compartment. Percent of total time spent in the light zone and in the dark zone. Error bars: mean + SEM. *, *p* < 0.05; **, *p* < 0.01 by Student *t* test. The number of mice per experimental group: 11 CT male, 14 CT female, 12 E3 male, 10 E3 female

To analyze the motor activity and anxiety level of mice, we used an open field task test. A mouse was placed in the center of a box and the activity of every mouse was recorded by a video recorder. The time spent, number of entries, and total distance travelled in the central area were analyzed. Our results show that E3 mice spent more time, made more entries to the central area, and travelled more distance in the central area, than CT mice at the age of 1 and 6 months male and female mice. There was no difference in the total distance travelled through the entire area (Fig. 4A). We also performed the light/dark transition test to measure anxiety-like behavior in mice. The E3 mice spent longer duration times and greater percentage of total time in the light area, and less duration times and percentage in the dark area than control mice in both male and female (Fig. 4B). These studies suggest that E_3_ may reduce anxiety but has no effect on locomotor activity.

### The impact of fetal E_3_ exposure on subsequent adult gene expression and methylation in the central nervous system

**Fig. 5:**
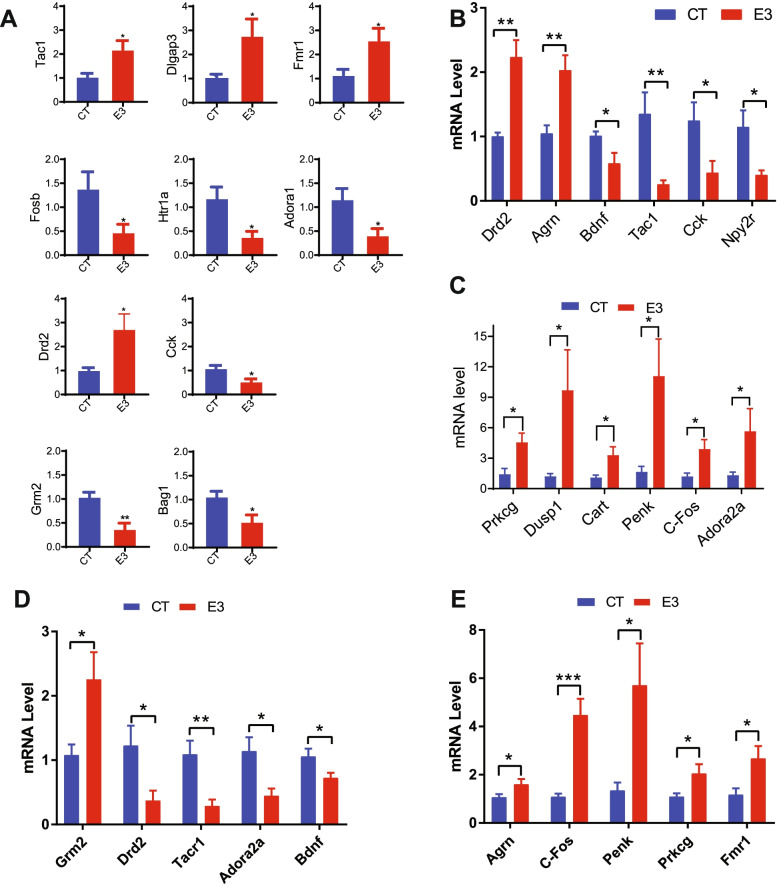
Prenatal exposure to E_3_ alters mRNA expression of anxiety-related genes in the adult offspring cortex. Eight-week-old pregnant female CD-1 mice were treated with vehicle DMSO (CT) or E_3_ (E3). At 6 months after birth, the gene expression in the offspring cortices were assessed by microarray and anxiety-related genes were validated by quantitative RT-qPCR. **A** Quantification of anxiety-related genes in the female offspring hippocampus, **B, C** in the male offspring cortex, and **D, E** female offspring cortex. Error bars: mean + SEM. *, *p* < 0.05; **, *p* < 0.01; ***, *p* < 0.001 by Student *t* test. *N* = 10/group

**Fig. 6:**
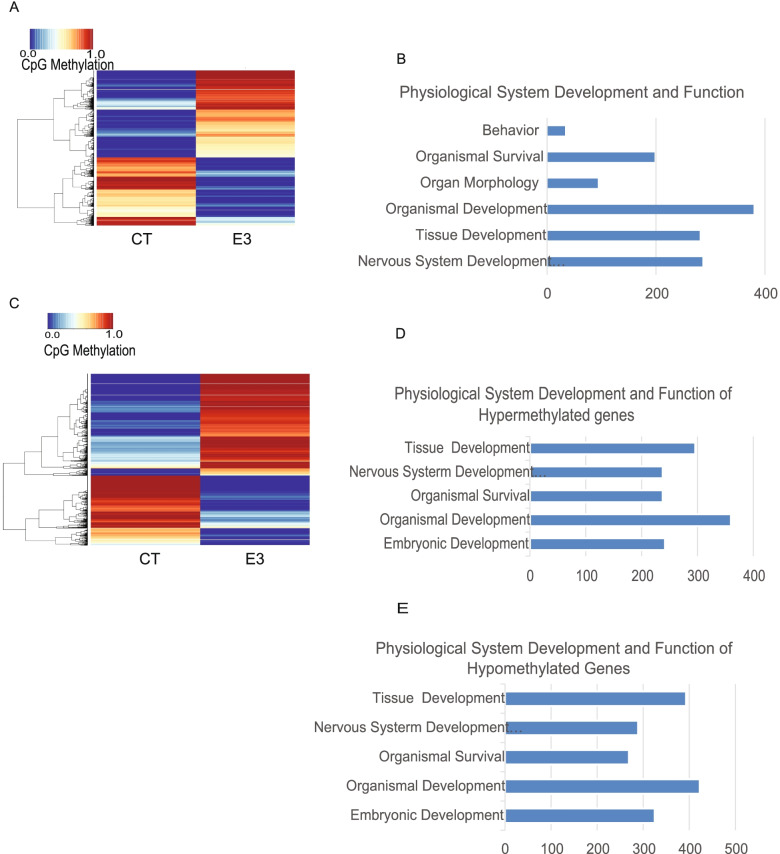
Prenatal exposure to E_3_ alters gene methylation in the adult offspring brain. **A** Heat maps showing the top 3000 differential CpG sites with altered methylation in E_3-_treated compared to control frontal cortex. **B** Grouping top 3000 genes according to physiological system development and function using the Ingenuity (IPA) software. **C** Heat maps of top 3000 CpG sites with altered methylation in hippocampus. Grouping of top 2000 of **D** hypermethylated and **E** hypomethylated differential CpG sites according to physiological system development and function using Ingenuity

Both ERα and ERβ are expressed by all neural cells [19]. In rodents, the ERα isoform is predominantly expressed in the preoptic area, most of the hypothalamic nuclei and the hippocampus, while ERβ is predominant in the olfactory bulb and cerebral cortex [20]. Since the frontal cortex and hippocampus play an important role in anxiety and stress responses, we performed an mRNA microarray of these tissues to investigate if prenatal exposure to E_3_ decreases the anxiety-like behavior by regulating anxiety-related gene expression. I total, 454/239 genes in the male cerebral cortex and 942/459 genes in the female cerebral cortex were up-/downregulated, respectively. A total of 360/238 genes in the male hippocampus and 1311/692 genes in female hippocampus were up-/downregulated (Additional file 2: Table S7 A-D). Overall, the female mice had more genes with altered expression than male mice. We next analyzed the biofunctions of the genes with altered expression. In the female hippocampus, 224 genes were related to behavior, 91/75/46 genes were related to locomotion/emotional/anxiety, and 44 genes were downstream of ERβ (Additional file 1: Fig. S6 A & B). Several of the genes related to anxiety and downstream of ERβ were validated by RT-qPCR (Fig. 5A). In the male hippocampus, 82 genes were related to nervous system development and function, and 16 genes downstream of ERα (Additional file 1: Fig. S6 C & D ). We also performed Qiagen Ingenuity Pathway Analysis (IPA) analysis for the genes where expression was influenced by E_3_ in the cortex. In the male cortex, 92 genes were related with behavior and 40/34 genes were related to locomotion/emotional behavior; there were 22 genes related with anxiety-like behavior, 18 genes were downstream of ERβ (Additional file 1: Fig. S6 E&F). Some anxiety-related genes downstream of ERβ were further verified by RT-qPCR (Fig. 5B,C). In female cortex, 142 genes were related with behavior and 55/42 genes were related to locomotion/emotional behavior; 31 genes were known downstream targets of ERβ (Additional file 1: Fig. S6 G & H). Some anxiety-related genes downstream of ERβ were further verified by RT-qPCR (Fig. 5D, E). These results suggest that fetal E_3_ exposure regulates anxiety-related gene expression through ERβ signaling. We also examined global gene methylation profile of the prefrontal cortex and hippocampus. In the prefrontal cortex, methylation of multiple CpG sites was altered (Additional file 2: Table S8). Figure 6A shows the top 3000 differential CpG sites with altered methylation in E_3-_treated compared to control frontal cortex, with 285 genes involved in nervous system development and function (Fig. 6B). Figure 6C shows the top 3000 CpG sites with altered methylation in the hippocampus. The top 2000 CpG sites that were hypermethylated (Additional file 2: Table S10A) include 238 genes involved in nervous system development and function (Fig. 6D). The top 2000 CpG sites that were hypomethylated (Additional file 2: Table S10B) include 289 genes involved in nervous system development and function (Fig. 6E). These results suggest that prenatal E_3_ exposure affects gene expression and methylation in the central nervous system.

### Fetal E_3_ exposure decreases DRD2 neuron activity and upregulates c-FOS expression in the frontal cortex in female offspring

**Fig. 7:**
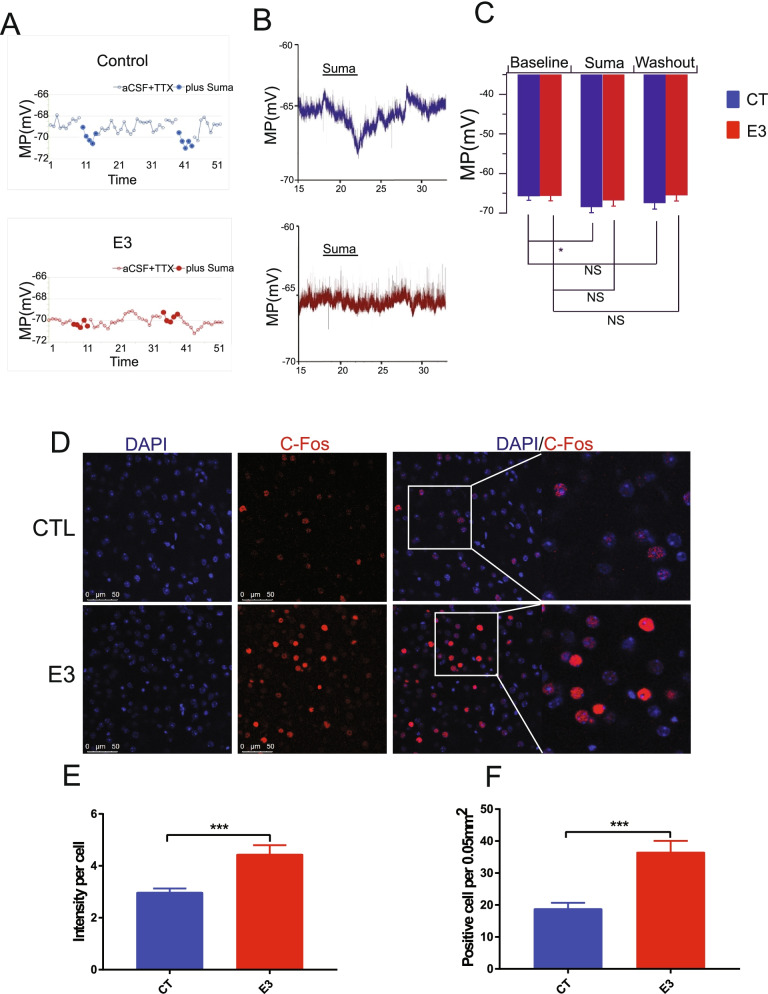
Prenatal exposure to E_3_ altered the effect of DRD2 agonist sumanirole (2 μM) on membrane potential (MP) in layer 2/3 pyramidal cells and c-Fos expression in the prefrontal cortex. **A–C** Perforated and whole-cell patch clamp recording. **A** Time courses showing the effect of sumanirole on MP in representative pyramidal cells from control (CT, top trace) and E_3_-treated (E3, bottom trace) mice. X-axis unit = minute; Y-axis unit = mV. The entire time course includes baseline (aCFS+TTX), sumanirole application and washout of sumanirole. **B** Two sample whole-cell recording traces. **C** Pooled results showed that MP was hyperpolarized significantly in control mice (blue bars) but not E_3_-treated mice (red bars). * *P* < 0.05, one-way ANOVA; ns *P* > 0.05, one-way ANOVA). **D** Immunofluorescence staining for c-Fos in the frontal cortex of the E_3_-treated and vehicle (DMSO) control group (CT). c-Fos expression in the front cortex is upregulated in the E_3_-treated female offspring. **E** The c-Fos fluorescence intensity per cell acquired by using image J. **F** The number of c-Fos-positive cells per 0.05 mm^2^. Error bars: mean + SD. ***, *p* < 0.001 by Student *t* test. *N* = 13/group

Among the genes with altered expression after E_3_ treatment in the frontal cortex, D2 receptor (DRD2), a member of the dopamine receptor G-protein-coupled receptor family, plays an important role in the modulation of locomotion, reward, reinforcement, and memory and learning. DRD2 was decreased by 5-fold in the cerebral cortexes of female offspring with E_3_ exposure (*N* = 12, *P* < 0.05). To examine whether DRD2 downregulation induces any functional consequences in E_3_-exposed offspring, we performed whole-cell patch clamp recording to examine the effects of a DRD2 agonist sumanirole (2 μM) on membrane potential (MP) in layer 2/3 pyramidal cells in the prefrontal cortex in the presence of voltage-dependent sodium channel blocker tetrodotoxin (TTX, 0.5 μM). In control mice, MP was hyperpolarized from a baseline of − 65.7 ± 1.1 mV (*n* = 14) to − 68.5 ± 1.4 mV (*n* = 14) during the application of sumanirole and recovered to − 67.5 ± 1.5 mV (*n* = 14) after the removal of sumanirole. The hyperpolarization was significant [F (2, 26) = 4.966, *P* < 0.05, one-way ANOVA]. In E_3_-treated mice, MP was − 65.6 ±1.2 mV (*n* = 15), − 66.8 ± 1.4 mV (*n* = 15), and − 65.5 ± 1.5 mV (*n* = 15) before, during, and after the application of sumanirole. MP was not significantly altered by sumanirole [F (2, 28) =1.978, *P* = 0.157, one-way ANOVA] (Fig. 7A–C).

Among the verified genes, c-Fos mRNA expression was upregulated by over 4-fold in the E_3_-treated mice as compared with the controls (Fig. 5C, E, *P* < 0.05) in both the male and female offspring. In agreement with mRNA expression result, c-Fos protein was increased by 2 fold in the E_3_-treated group compared to the control group (Fig. 7D–F, *P* < 0.0001).

### E_3_ treatment impacts formation of ER-epigenetic modifiers complex and their binding to target genes

**Fig. 8:**
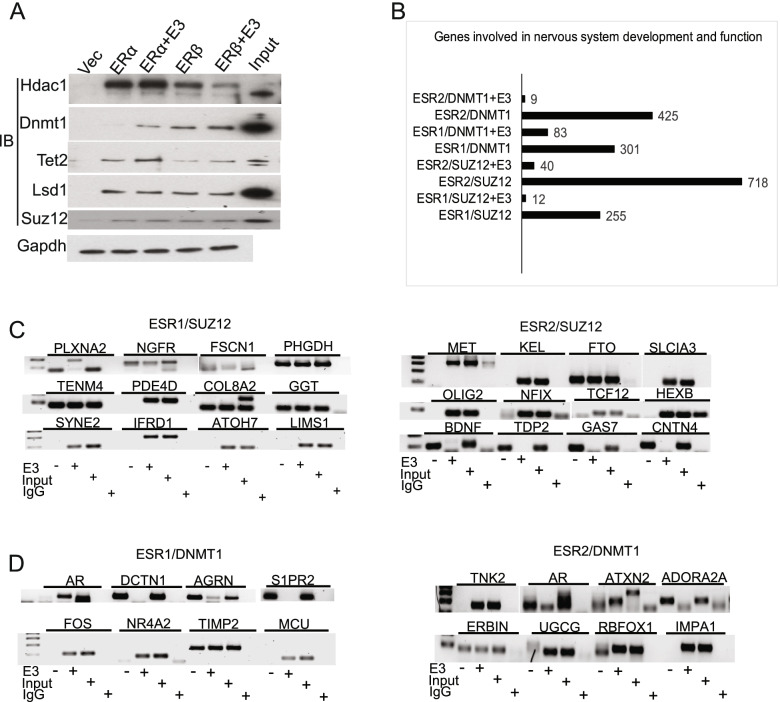
E_3_ treatment impacts formation of ER-epigenetic modifiers complex and their binding to target genes in neurons. Neuroblastoma SH-SY5Y cells were transfected with the Flag chimera expressing plasmids (Vec), Flag-ERα, or Flag-ERβ respectively, and then treated with/without E_3_. **A** Co-immunoprecipitation (IP) of ERα/β and DNA/histone modifiers with an anti-Flag antibody followed by immunoblotting (IB) using the indicated antibodies. The input indicates the expression level in the whole cell lysate before IP. **B, C** Neuroblastoma SH-SY5Y cells were treated with/without E_3_ (CT, E_3_), and **B** ChIP (chromatin immunoprecipitation) was performed using an anti-estrogen receptor α/β (ESR1/ESR2) antibody (First ChIP) and then with an anti-SUZ12 or DNMT1 antibody (Re-ChIP). The bound DNA was then identified by sequencing. The chart shows the numbers of genes involved in the nervous system development and function. **C, D** Validation of the target DNA sequences from **B** by Chip-PCR. The data are representative of three independent experiments. Uncropped blots available in Additional file 3: Fig. S7

After finding that E_3_ influences uterine gene expression by regulating epigenetic modifier-ER-DNA interactions (Additional file 2: Table S2), we asked if E_3_ also regulates ER-DNA epigenetic interaction in neurons. We performed immunoprecipitation and Western blotting to identify the interacting proteins of ERα and ERβ in a neuroblastoma cell line, SH-SY5Y. Indeed, both ERα and ERβ co-precipitated with several DNA/histone modifiers including HDAC1, SUZ12, LSD1, DNMT1, and TET2. Interestingly, E_3_ treatment modestly enhanced the interaction between ERs and DNMT1 or TET2 (Fig. 8A).

We performed a Re-ChIP assay to further confirm that E_3_ activates ER binding to DNA modifiers, which together bind to their target genes. We identified 2391 DNA promotor sites that were bound by both ERα and DNMT1 simultaneously, 53 sites were bound by both ERβ and DNMT1, 253 sites were bound by both ERα and SUZ12, and 260 sites were bound by both ERβ and SUZ12, after E_3_ treatment (Additional file 2: Table S9 A-D). Interestingly, E_3_ treatment decreases binding activity of the ER/DNMT1, as well as the ER/SUZ12 to their target genes, which have a role in nervous system development and function (Fig. 8B). We verified several of these genes by Chip-PCR (Fig. 8C & D). The altered expression of four anxiety-related genes, c-Fos, Adora2a, BDNF, and AGRN were additionally confirmed by RT-qPCR (Fig. 5B–E).

## Discussion

Estriol (E_3_) is by far the most abundant estrogen in pregnant women and fetuses, though its potency is considered the weakest among the three major estrogens (estradiol, estrone, and estriol) [1, 2]. However, the impact of the high but transient gestational levels of E_3_ on fetal development as well as on subsequent adult health and disease is unknown. We have identified that exposure of fetal mice, which do not normally produce significant levels of E_3_, to this estrogen improved adult offspring reproductive success and behavior by epigenetically programming the fetus.

In nature, without dramatic increases of E_3_ in pregnancy, rodents still reproduce successfully, though with a high incidence of embryo resorption, premature delivery, post-term labor, and stillbirth. Interestingly, fetal exogenous E_3_ treatment significantly improved the chance of pregnancy, reduced the incidence of resorption, and eliminated premature/post-term delivery/stillbirth in prenatally exposed female offspring. Moreover, E_3_ treatment had marked neurological effects, notably reduced the anxiety levels of both male and female offspring, accompanied by a significant decrease in DRD2 expression and activity. Intriguingly, DRD2 is the primary target for both typical and atypical antipsychotic drugs and for drugs used to treat Parkinson’s disease [21]. These results suggest that transient gestational E_3_ exposure has a long-term beneficial impact on offspring health. This lifetime impact likely results from epigenetic modifications. Indeed, the mRNA levels of several hundred genes were up- or downregulated in the adult uteri and brains of fetal E_3_-treated mice and approximately two thousand genes were either hyper- or hypo- methylated in the uteri and brains compared to the control mice. Mechanistically, we demonstrated that E_3_ affects both the complexing of nuclear ERs with several DNA/histone modifiers, as well as the binding of these complexes to target genes in a manner distinct from estradiol. Additionally, E_3_ could also activate membrane estrogen receptors as evidenced by enhanced AKT phosphorylation following E_3_ treatment. The biological pathways of the affected genes are beyond that of classic estrogen action on estrogen sensitive genes, suggesting an extensive influence of E_3_ on the offspring. Interestingly, in our study, many more genes showed altered methylation than differential expression induced by E_3_ exposure. This implies that not all methylation is of functional significance. Further, not all of the genes that showed altered expression were methylated, suggesting an indirect effect of methylated genes on downstream targets or a role for the histone modification identified here.

Because of its varying binding affinity for ERα and ERβ and varying expression levels of ERs in different tissues, each estrogen and estrogen combination mediate variable downstream effects that are both concentration- and tissue context-dependent [22]. Compared to E_2_, E_3_ binds weakly to both ERs, with a preference for ERβ [23]. In humans, when E_3_ becomes dominant during pregnancy, it may synergize with E_2_ to stimulate estrogenic responses, but could also inhibit E_2_ signaling by competing for the same ER binding site [24]. However, the effect of E_3_ on epigenetic programming is potent and distinct from E_2_. Moreover, gestational exposure to E_3_ can alter adult offspring responsiveness to E_2_, exemplified by changes in the mRNA levels of 114 genes in the uteri of E_3_-E_2_ mice compared to CT-E_2_ mice. These observations further suggest an effect of E_3_-mediated epigenetic programming on subsequent E_2_ response, programming estrogen sensitivity.

Epigenetic modification is dynamic, beginning from the developing germ cells and continuing throughout life [25]. This process involves the action of many DNA and histone modifiers. This study has revealed novel complex interactions between E_3_, ERs, and epigenetic modifiers, which may underlie changes in global gene expression and DNA methylation profile. Treatment with E_3_ increased the protein levels of epigenetic modifiers DNMT1, EZH2, SUZ12, LSD1, and CBP in the offspring uteri. DNMT1 maintains existing DNA methylation patterns [26]. We have previously shown that fetal exposure to diethylstilbestrol (DES), a well-known human reproductive tract morphogen, increased the expression of DNMTs, which resulted in permanent changes in the DNA methylation profile of the uterus [11]. EZH2 functions as the catalytic subunit of the Polycomb Repressive Complex, which methylates K27 of histone 3 (H3), leading to long-term gene silencing. We have also shown that DES and bisphenol A (BPA) increases the expression of EZH2, which led to alterations in histone methylation and subsequent gene silencing in the mammary gland [14]. SUZ12 is required for the catalytic function of EZH2 in the Polycomb Repressive Complex and is also involved in the E_3_-mediated epigenetic changes described here. LSD1 is a histone demethylase that can function as a transcriptional deactivator by demethylating K4 of Histone 3, or a transcriptional activator by demethylating K9 of Histone 3. Its ability to activate transcription is triggered by the presence of ERα at the chromatin [27, 28]. LSD1 is also involved in the demethylation of DNMT1, which ensures its stability [29]. In addition to ER effects on epigenetic modifier expression, we found that HDAC1, LSD1, TET2, DNMT1, and SUZ12 all directly interacted with ERα. E_3_ altered the interaction of SUZ12, LSD1, and DNMT1 with ERα in endometrial cells and altered the interaction of TET2 and DNMT1 with ERα and ERβ in neuroblastoma cells. The Re-ChIP assay further validated that E_3_ treatment modulated binding of ER-DNA modifier complex to their target genes in neuroblastoma cells. Thus, E_3_ treatment influences formation of ER-epigenetic modifier complexes and their binding to target genes.

E_2_ binds cytoplasmic ERα and ERβ, which then translocate into the nucleus and bind to estrogen response elements (ERE) on chromosomal DNA together with histone acetyltransferases and other co-regulators to regulate gene transcription [30]. In addition, some ER localizes to the endoplasmic reticulum and plasma membrane and may trigger rapid non-genomic actions of estrogens [31–33], leading to transcriptional and translational regulation of target genes [30]. In utero exposure to a synthetic estrogen, diethylstilbestrol (DES), increases the expression of EZH2, a member of Polycomb repressive complex 2 (PRC2) that methylates H3K27 [14], thus promoting long-term gene silencing [34]. For example, DES permanently alters homeobox A10 (HOXA10) gene expression by epigenetically modifying the HOXA10 promoter [11, 13]. While DES is a potent estrogen, the endocrine disruptor bisphenol A (BPA) is a weak estrogen that can also impact uterine gene expression [35], and modify DNA methylation [12, 36]. Interestingly, the methylation pattern induced by BPA is distinct from that seen after DES exposure. Taken together, these data imply that several estrogens, including those classically considered of low potency, can alter epigenetic programming of gene expression.

Synthetic estrogens such as DES or tamoxifen are known to induce distinct conformational changes in ERs [37–40]. Similarly, selective estrogen receptor modulators (SERMs) also induce unique ER conformational changes [41–43]. These conformational changes differentially regulate binding of co-activator or co-repressor proteins that in turn control ER target gene transcription. Classically, estrogen potency has been described by the ability to induce expression of known estrogen-responsive genes and a classic estrogenic physiologic response in adults [44]. Here we show that the ability of ERs to bind epigenetic modifying proteins varies with exposure to different ligands. However, the ability of estrogen ligands to induce ER binding of epigenetic modifiers is not proportional to the estrogenic induction of ER binding to classical co-regulators or known adult estrogenic responses. The conformational changes induced in ER relevant to epigenetic modifier binding are likely in areas distinct from those used to bind classic co-regulators. Thus, the ability to bind these epigenetic modifiers would be independent of their potency as regulators of classical estrogen action. This likely underlies the significant role of E_3_ in epigenetic programming of fetuses, through estrogen-type-dependent induction of distinct conformational changes in ERs that allow interaction with specific epigenetic modifiers. This may also broadly explain why endocrine disruptors are often weak estrogens as measured by classic estrogen responses, yet may induce significant changes in exposed animals and humans out of proportion to their predicted potency.

## Conclusions

Our studies reveal a profound and potent role of gestational E_3_ in epigenetically programming the fetus, which promotes adult offspring reproductive and mental health. Primates have evolved a common signaling mechanism to allow enhancements in the brain and reproductive tract that are unique to these species. Since the biological pathways affected by E_3_ are extensive, future work will examine its role in other organ systems and disease models.

## Methods

### Animal care and treatment

CD-1 mice were obtained from Charles River Laboratories (Wilmington, MA, USA). Eight-week-old CD-1 female mice were bred to male mice of the same strain and examined every 12 h until the presence of a vaginal plug was detected. Detection of a vaginal plug was considered day 0.5 of pregnancy. Eight pregnant CD-1 mice were then housed individually and continuously treated with E_3_ (50 μl of 0.46 mg/ml in DMSO to approximate human exposure) or vehicle control (DMSO) via a mini-osmotic pump (Alzet model # 1002) beginning on day 9 of gestation. This timing of E_3_ treatment was based on the kinetics of E_3_ production during human pregnancy. The level of E_3_ is considerably increased from week 14 after pregnancy (second trimester), we thus chose a date (Day 9) within the “second trimester.” For reproduction experiments, at the age of 8 weeks, the E_3_- and mock-treated female mice were bred to untreated male CD-1 mice. The incidence of mating (vaginal plug), pregnancy, fetal resorption, stillbirth, the dates of delivery, and litter size were monitored. The pregnancy rate was defined as the percentage of pregnant mice out of the initial total female mice. The resorption rate was defined as the ratio of the pregnant mice who failed to give birth to the total pregnant mice. For E_2_ responsiveness experiments, at the age of 6 weeks, the E_3_ and mock-treated female offspring (*N* = 12 per group) were ovariectomized; after 2 weeks, they were treated with a single intraperitoneal injection of either 300 ng of estradiol (E_2_) or vehicle control (DMSO). Six hours after injection, the mice were euthanized by cervical dislocation under inhaled CO_2_ anesthesia. Uteri were harvested for mRNA, genomic DNA, and protein analyses. We performed three independent experiments, with 12 animals/group each time. We began each experiment by treating pregnant mice with E_3_ (12 mice/group), and ended by analyzing their offspring (both fetuses and adults, totaling 143). The size of each litter varied. Ninety-five fetuses were used for reproduction experiments, 48 for behavioral tests. Animals were housed in a 12-h day/night cycle. All experiments were conducted in accordance with Yale University Animal Care Committee Guidelines.

### Behavioral tests

The open field test was performed to assess the degree of anxiety and locomotor activity in mice [45]. E_3_-treated offspring and controls mice (*N* = 24 per group) underwent open field-testing at the age of 1 month and 6 months. The open field test arena is an open square box (50 × 50 × 40 cm) composed of a uniform clear floor without bedding. The box is virtually demarcated into a central zone and peripheral zones. An experimental mouse was placed in one corner of the box and allowed to explore the area for 20 min. The overall activity of the mouse in the box was recorded by a computer-based video recorder. The time spent, number of entries, and total distance travelled in the central zone were analyzed. The mice with a higher anxiety level tend to spend more time in the periphery and less time in the central area. The overall distance travelled by each mouse was considered an indicator of locomotor activity.

The light/dark transition test is another widely used test to measure anxiety-like behavior in mice. The number of entries into the bright chamber and the duration of time spent there are indices of bright-space anxiety in mice [46]. The light/dark transition test consists of a cage (21 × 42 × 25 cm) divided into two chambers of equal size. Behavioral testing was performed between 9:00 A.M. and 6:00 P.M. Mice were transferred to the behavior testing room 30 min before the first trial began. One chamber was darkly illuminated by red diodes (390 lux), whereas the other chamber was white light (2 lux). A mouse was placed into the cage and allowed to move freely between these two chambers for 5 min, monitored by an infrared camera. The software used for data acquisition and analysis (Image LD4) is based on the public domain ImageJ program (developed by Wayne Rasband at the National Institute of Mental Health and available at http://rsb.info.nih.gov/ij/), which was modified by Tsuyoshi Miyakawa (available through O'Hara & Co., Tokyo, Japan). The time spent in each chamber, and the rate of latency to enter the light and dark chambers were recorded by Image LD4. After each trial, all chambers were cleaned with super hypochlorous water to prevent a bias based on olfactory cues [47].

The Novel Object Recognition (NOR) task is used to evaluate cognition, particularly recognition memory, in rodent models. This test is based on the spontaneous tendency of rodents to spend more time exploring a novel object than a familiar one. The choice to explore a novel object demonstrates memory and the ability to learn. Mice were individually habituated to an open field box (21 × 42 × 25 cm) for a training session and retention session. During the training session (on day 1), two novel objects were placed into the open field and the animal was allowed to explore for 3 min. The time spent exploring each object was recorded. During the retention session (on day 3), the animal was placed back into the same box, in which one of the familiar objects used during training was replaced by a novel object and allowed to explore freely for 3 min. A preference index, the amount of time spent exploring any one of the two objects (training session) or the novel one (retention session) was used to measure recognition memory [48].

The Morris water maze was used to conduct the reference memory task, probe test, and working memory task for evaluation of long-term memory, spatial learning and memory, and short-term memory abilities of mice, respectively, as previously described [49] with slight modifications. Briefly, the test was performed in a circular water pool (diameter 152 cm; height 30 cm). The pool was divided into 4 quadrants (I, II, III, and IV) and the water was kept at room temperature (22 ± 2 °C). Track path length, escape latency, and time spent in each quadrant were monitored using an overhead camera during the experimental period.

In vitro electrophysiology, coronal brain slices containing prefrontal cortex, 300 μm thick, were cut from mice as reported previously by us [45]. Briefly, mice were deeply anesthetized with isoflurane and decapitated; brains were then rapidly removed, trimmed to a small tissue block, and sectioned with a vibratome in an oxygenated (with 5% CO_2_ and 95% O_2_) cutting solution at 4 °C containing (in mM): sucrose 220, KCl 2.5, CaCl_2_ 1, MgCl_2_ 6, NaH_2_PO_4_ 1.25, NaHCO_3_ 26, and glucose 10, pH 7.3 with NaOH. After preparation, slices were maintained in a storage chamber with oxygenated artificial cerebrospinal fluid (ACSF) containing (in mM): NaCl 124, KCl 3, CaCl_2_ 2, MgCl_2_ 2, NaH_2_PO_4_ 1.23, NaHCO_3_ 26, glucose 10, pH 7.4 with NaOH. Prefrontal slices were transferred to a recording chamber constantly perfused with ACSF at 34 °C with a rate of 2 ml/min after at least 1 h recovery. Both perforated and whole-cell current clamp were performed to observe membrane and action potential with a Multiclamp 700A amplifier (Axon instrument, CA, USA) as described previously [50, 51]. Spontaneous action potentials were blocked by 0.5 μM tetrodotoxin (TTX) and membrane potential recorded in the neurons under current clamp. We recorded for approximately 20 min for baseline at a glucose concentration of 2.5 mM. When the membrane potential was stable, the D2 agonist, sumanirole 2 μM was added in for 5 min, followed by recovery using control solution for at least 15 min. The patch pipettes with a tip resistance of 4–6 MΩ were made of borosilicate glass (World Precision Instruments, FL, USA) with a Sutter pipette puller (P-97) and filled with a pipette solution containing (in mM): K-gluconate 135, MgCl_2_ 2, HEPES 10, EGTA 1.1, Mg-ATP 2, Na_2_-phosphocreatine 10, and Na_2_-GTP 0.3, pH 7.3 with KOH. After a giga-ohm (GΩ) seal and whole-cell access were achieved, the series resistance (between 10 and 20 MΩ) was partially compensated by the amplifier. All data were sampled at 10 kHz and filtered at 3 kHz with an Apple Macintosh computer using AxoGraph X (AxoGraph, Inc, Australia.). Electrophysiological data were analyzed with AxoGraph X and plotted with Igor Pro software (WaveMetrics, 1OR, USA).

### Gene expression analysis

The tissue intended for mRNA and gDNA analysis was placed in 1 mL TRIzol per 100 mg tissue (Invitrogen, Carlsbad, CA, USA). Tissue was homogenized on ice, and total RNA was isolated using methodology described by the manufacturer. Total RNA was divided into two pools/group (each pool from 6 mice) and was then labeled and hybridized to a MouseRef-8 v2.0 Expression BeadChip kit (Illumina Inc., CA, USA) to profile the whole-genome expression by the Keck Biotechnology Resource Laboratory at Yale School of Medicine. Data was analyzed by MEPH Biostatistics using Genome Studio Data analysis software. Data was normalized, and genes showing significant positive or negative changes (more than 1.5-fold) versus control were identified. Expression of selected genes was verified by real-time RT-PCR as described below.

### Quantitative RT-PCR

Total RNA (500 ng) was reverse transcribed in a 20 μl of reaction mixt using the iScript cDNA synthesis kit (Bio-Rad, Hercules, CA, USA). The reaction mix was incubated for 5 min at 25 °C, 30 min at 42 °C, and 5 min at 85 °C using an Eppendorf Mastercycler (Eppendorf North America). Quantitative real-time RT-PCR reactions were prepared using the iQ SYBR Green Supermix (Bio-Rad). Each PCR reaction consisted of the following: 5 μl of cDNA template, 0.5 μl of forward primer (10 μM), 0.5 μl of reverse primer (10 μM), 4 μl of nuclease-free H_2_O, and 10 μl of iQ SYBR Green Supermix. β-Actin was used as a housekeeping gene. The β-Actin primers have been described previously [35]. Primer sequences are listed in supplemental table 1.

The Bio-Rad iCycler iQ system (Bio-Rad) was used to quantify fluorescence of PCR products during amplification. RT-qPCR reactions were performed for 40 cycles at 95 °C for 15 s and 60 °C for 15 s, and 72 °C for 15 s. Relative gene expression was determined by analyzing data using the 2^−ΔΔCT^ method to adjust for expression of β-actin for uterus; and PPIA for brain. Specificity of the amplified products and the absence of primer-dimmers were confirmed by melt curve analysis. All products obtained yielded the predicted melting temperature. All experiments were conducted in triplicate. Samples without a cDNA template were used as negative controls.

### DNA methylation array

Genomic DNA was extracted using the Qiagen DNeasy Kit, following the manufacturer’s protocol including the RNase A treatment. Genomic DNA was then digested into 200–1000 bp fragments by Mse I restriction enzyme (New England Biolabs). Immunoprecipitation (IP) of methylated genomic DNA was performed with a mouse monoclonal anti 5-methylcytidine antibody (Abcam, #10805). IP and input DNA were amplified with WGA2 Kit (Sigma-Aldrich). The amplified IP and input DNA were purified using the Qiagen QIAquick PCR Purification Kit (Catalog No. 28106) according to the manufacturer’s protocol. The IP and input gDNA were then labeled, hybridized, and scanned by the Keck Biotechnology Resource Laboratory using the Mouse DNA Methylation 3×720K CpG Island Plus RefSeq Promoter Array [Methylated DNA Immunoprecipitation (MeDIP)] (Nimble Gen). Data were analyzed by using Roche NimbleGen Software DEVA. Genes presenting a peak score greater than 3 were identified.

### Methylation mini-sequencing

Mouse hippocampus and frontal cortexes were removed and DNA was extracted using the Quick-DNA Microprep Kit (Cat#D3020) from Zymo research. Samples were sent to Zymo Research to process and analyze using the Methyl-MiniSeq Full Service library preparation, sequencing, and bioinformatics pipeline. Briefly, libraries were prepared from 200 to 500 ng of genomic DNA digested with 60 units of TaqαI and 30 units of MspI (NEB) sequentially and then extracted with Zymo Research (ZR) DNA Clean & Concentrator TM-5 kit (Cat#: D4003). Fragments were ligated to pre-annealed adapters containing 5’-methylcytosine instead of cytosine according to Illumina’s specified guidelines (www.illumina.com). Adaptor-ligated fragments of 150–250 bp and 250–350 bp in size were recovered from a 2.5% NuSieve 1:1 agarose gel (ZymocleanTM Gel DNA Recovery Kit, ZR Cat#: D4001). The fragments were then bisulfite-treated using the EZ DNA Methylation-Lightning TM Kit (ZR, Cat#: D5020). Preparative-scale PCR was performed and the resulting products were purified (DNA Clean & ConcentratorTM - ZR, Cat#D4005) for sequencing on an Illumina HiSeq. Sequence alignments and data analysis: Sequence reads from bisulfite-treated Methyl-MiniSeq® libraries were identified using standard Illumina base calling software and then raw FASTQ files were adapter, filled-in nucleotides, and quality trimmed using TrimGalore0.6.4. FastQC0.11.8 was used to assess the effect of trimming and overall quality distributions of the data. Alignment to the mm10 reference genome was performed using Bismark0.19.0. Methylated and unmethylated read totals for each CpG site were called using MethylDackel0.4.0. The methylation level of each sampled cytosine was estimated as the number of reads reporting a C, divided by the total number of reads reporting a C or T. Fisher’s exact test was performed on each CpG, CHG, and CHH site that has at least 5× coverage. Ten percent difference in methylation value between groups and a *p*-value (unadjusted) of < 0.05 was used to identify significantly differentially methylated cytosines. Promoter, gene body, and CpG island annotations were added for each CpG included in the comparison where available.

### Cell culture

Ishikawa cells were maintained in MEM (Sigma-Aldrich, MO), SH-SY5Y cells were maintained in DMEM/F12 (Sigma-Aldrich, MO) both containing 10% fetal bovine serum (FBS) and 1% penicillin/streptomycin. For estrogen treatment, Ishikawa cells and SH-SY5Y cells were maintained in phenol red-free MEM (Sigma Chemical) or phenol red-free DMEM/F12 containing 10% (vol/vol) charcoal-stripped calf serum, 1% penicillin/streptomycin, and 1% sodium pyruvate. Plasmid-transfected cells (4 × 10^7^ Ishikawa cells and 4 × 10^6^ SH-SY5Y cells) were seeded to a 15-cm dish and incubated at 37 °C, 5% CO_2_ for 6 h. Media were changed to serum-free media for 12 h. The Ishikawa cells (~70% confluence) were subsequently treated with E_2_, E_3_, bisphenol A (BPA) diethylstilbestrol (DES) (1 × 10^−6^ M) or dimethyl sulfoxide (control) for 6 h; and SH-SY5Y cells (~70% confluence) were subsequently treated with E_3_ for 24 h. Cells were homogenized for immunoprecipitation and chromatin immunoprecipitation.

### Plasmid construction, cell transfection, and immunoprecipitation

The human ERα cDNA was amplified by PCR using primers: forward 5′-TTGGATCCATGACCATGACCCTCCACACCAA-3′ and reverse 5′-TCGAATTCTCA***TTTATCGTCATCGTCTTTGTAGTC***GACCGTGGCAGGGAAACCCTC -3′ (reverse primer contains the flag sequence in bold). PCR program was as follows: 95 °C for 60 sec, 59 °C for 60 s, and 72 °C for 90 s for 35 cycles. *Flag-ERα* cDNA was cloned into the BamHI and EcoRI site of pcDNA3.1(+) (Invitrogen). Flag-pcDNA3.1(+) without the *ERα* insert was used as a control (Invitrogen). Flag-ERβ were obtained from Addgene (plasmid #35562). (4 × 10^7^) Ishikawa cells and (4 × 10^6^) SH-SY5Y cells were treated with transfection mix [Lipofectamine 2000 (Invitrogen) mixed with 40 μg of either pcDNA 3.1(+)/*Flag-ERα* or pcDNA /*Flag-ERβ* or pcDNA3.1(+)/Flag] for 20 min. Cells were plated into a 15-cm dish and incubated at 37 °C, 5% CO_2_ for 6 h. Fresh phenol red-free and serum-free medium were added, and cells were incubated for an additional 12 h, then treated with E_2_, E_3_, or bisphenol A (1 × 10^−6^ M) for 24 h. Cells were lysed, bound cellular proteins were purified using an anti-Flag antibody cross-linked to agarose beads, washed, and eluted with 3xFlag peptide according to the manufacturer’s product manual (Sigma-Aldrich).

### Tandem purification and MudPIT

The eluted Flag-ERα, Flag-ERβ, and its potential binding proteins were subject to Multi-Dimensional Protein Identification Technology (MudPIT) according to a published methodology [52]. Briefly, the ERα and ERβ protein complexes were isolated from cell extracts by immunoprecipitation with an α-Flag antibody (cross-linked to magnetic beads) according to established procedures with modifications. The bound proteins were eluted with Flag peptide and then digested with trypsin for the MudPIT analysis. The digested protein mixture was analyzed by an Agilent 1200 quaternary HPLC (Agilent, Palo Alto, CA) using a modified 11-step separation. As peptides were eluted, they were electrosprayed directly into a hybrid LTQ-Orbitrap mass spectrometer (Thermo Fisher Scientific, San Jose, CA) with the application of a distal 2.5 kV spray voltage. A cycle of one FT full-scan mass spectrum (400–1400 m/z, 60,000 resolution) followed by 8 data-dependent LTQ MS/MS spectra at a 35% normalized collision energy was repeated continuously throughout each step of the multidimensional separation. Application of mass spectrometer scan functions and HPLC solvent gradients were controlled by the Xcalibur data system (Thermo Fisher Scientific, San Jose, CA). MS/MS spectra were processed and searched with the ProLucid algorithm against the EBI human IPI database (ftp://ftp.ebi.ac.uk/pub/databases/IPI/, version 3.30) that was concatenated to a decoy database in which the sequence for each entry in the original database was reversed. ProLuCID results were assembled and filtered using the DTASelect (version 2.0) program. The MudPIT data from Flag-ERα, Flag-ERβ, and Flag control were compared. Proteins were designated as putative interacting proteins if they were identified only in Flag-ERα and Flag-ERβ samples but not in the Flag control, or the spectral count of each protein co-purified with Flag-ERα and Flag-ERβ were at least four times greater than that of the Flag control.

### Antibodies and Western blotting

Antibodies against pAKT, AKT, EZH2, DNMT1, HDAC1, GAPDH, and LSD1 were obtained from Cell Signaling Technology and used at a dilution of 1:2000; H3 (tri methyl k27), SUZ12, KAT8, and TET2 were purchased from Abcam and used at a dilution of 1:1000; ER-a [Santa Cruz (HC-20)] and CBP(R&D) were used at a dilution of 1:1000. Mouse tissues and culture cells were lysed in lysis buffer (1% Nonidet P-40, 50 mM Tris·HCl, 0.1 mM EDTA, 150 mM NaCl, proteinase inhibitors, and protein phosphatase inhibitors) and cleared by centrifugation at 10,000×*g* for 10 min at 4 °C. Equal amounts (20 μg) of whole tissue/cell lysates or immunoprecipitation samples were electrophoresed by SDS-PAGE and transferred to PVDF membranes. The membranes were incubated with appropriately diluted primary antibodies at 4 °C overnight or room temperature for 1 h. After three washes (10 min each), the blots were incubated with peroxidase-conjugated secondary antibodies at room temperature for 1 h. The blots were washed thoroughly (3 washes, 10 min each) and developed with enhanced (GE Health) or super sensitive (Pierce) chemiluminescence substrates.

### Histology and immunofluorescence

Thirteen uterine or brain cortex tissues/group were fixed in 4% paraformaldehyde, embedded in paraffin, and cut into 5-μM-thick slices. Slices were mounted on glass slides, deparaffinized, and stained with hematoxylin and eosin (H&E). For immunofluorescence staining, after antigen retrieval, samples were permeabilized and blocked with 0.5% Triton in Tris buffered saline, pH 7.4, and 5% normal donkey serum. After blocking, sections were incubated with primary antibodies [c-Fos (Santa Cruz, sc-52, 1:500)] overnight at 4 °C. Sections were washed three times, followed by an incubation with secondary antibodies (Invitrogen, 1:200) diluted in blocking solution and were incubated for 1 h at RT. After three washes, sections were mounted in a mounting medium with DAPI (Vectashield, H-1500) and visualized using a Leica SP5 Confocal Microscope.

### Re-ChIP sequencing

The Re-ChIP (chromatin immunoprecipitation) kit was purchased from Active Motif (kit #53016). Briefly, cells were fixed in 1% formaldehyde for 10 min and quenched with 0.125 M glycine. Chromatin was isolated by the addition of lysis buffer, followed by disruption with a dounce homogenizer. The DNA was sheared to an average length of 300–500 bp using an enzymatic shearing kit from Active Motif (kit #53035). The lysate was precleared with protein A agarose bead (Invitrogen, Waltham, USA) and Flag antibody (2 μg) directed against the DNA-binding protein Flag-ERα and Flag-ERβ. Protein-chromatin complexes were washed, eluted with elution buffer that prevents the majority of the first antibody from participating in the second IP reaction. The chromatin was then desalted, and a second ChIP step was performed using an anti- SUZ12 (2μg) or DNMT1 (2μg) antibody. The cross-link of these sequentially immunoprecipitated protein-DNA complexes was then reversed and subjected to RNase and proteinase K treatment. Reverse crosslinking was performed overnight at 65 °C. Then DNA was purified using a ChIP DNA purification kit from Active Motif (#58002) and submitted to Keck Biotechnology Resource Laboratory at Yale School of Medicine for sequencing. Briefly, approximately 5–10 ng of DNA is cleaned up using Ampure XP SPRI beads (Beckman Coulter Genomics). The DNA is then end-repaired, A-tailed, adapter ligated, and PCR enriched (8–10 cycles). Indexed libraries that meet appropriate cut-offs are quantified by both RT-qPCR using a commercially available kit (KAPA Biosystems) and insert size distribution determined with the LabChip GX. Samples with a yield of ≥0.5 ng/μl are used for sequencing. Flow Cell Preparation and Sequencing: Sample concentrations are normalized to 1 nM and loaded onto an Illumina HiSeq4000 flow cell at a concentration that yields 25–50 million passing filter clusters per sample. Samples are sequenced using 100-bp paired-end sequencing on an Illumina HiSeq 4000 according to Illumina protocols. The 10-bp dual index is read during additional sequencing reads that automatically follows the completion of read 1. Data generated during sequencing runs are simultaneously transferred to the YCGA high-performance computing cluster. A positive control (prepared bacteriophage Phi X library) provided by Illumina is spiked into every lane at a concentration of 0.3% to monitor sequencing quality in real time.

### Statistical analyses

The sample size chosen for our animal experiments in this study was estimated based on pilot data performing similar sets of experiments and power analysis. All animal results were included. All data were analyzed with GraphPad Prism software by nonparametric Mann-Whitney test, 2-tailed Student *t* test, one-way ANOVA, or multiple *t* tests, depending on the data distribution and the number of comparison groups. *P* values of less than 0.05 were considered statistically significant. The sample sizes (biological replicates) and specific statistical tests used for each experiment were detailed in each figure legend.

## Supplementary Information

**Additional file 1: Fig. S1** Prenatal exposure of estriol (E_3_) alters the global gene expression profile of the female offspring uteri. **A)** Grouping of altered genes in E_3_-treated animals compared to vehicle-treated groups according to biological functions using the Ingenuity (IPA) software. **B)** Pathway analyses using Ingenuity (IPA) software. **Fig. S2** A, B, C, D) Prenatal exposure of E_3_ results in significant hypomethylation of over 2000 genes in the female offspring uteri. Grouping of altered genes in E_3_-treated compared to vehicle-treated groups according to **A)** biological functions and **B)** molecular functions using Ingenuity (IPA) software. **C-D)** Pathway analyses using Ingenuity (IPA) software. **E, F**) E_3_ treatment increases the numbers of ER-SUZ12-bound genes. Ishikawa cells were treated with/without E_3_, and ChIP (chromatin immunoprecipitation) was performed using an **E)** anti-estrogen receptor α (ERα) or **F)** ERβ antibody (First ChIP) and then with an anti-SUZ12 antibody (Re-ChIP). The bound DNA was then identified by sequencing. The Venn diagrams show the numbers of genes bound by the ER-SUZ12 complex with/without E_3_ treatment. **Fig. S3)** Prenatal exposure to E_3_ results in significant hypermethylation of over 2500 genes in the female offspring uteri. Grouping of altered genes in E_3_-treated compared to vehicle-treated groups according to **A)** biological functions and **B)** molecular functions using the Ingenuity (IPA) software. **C-D)** Pathway analyses using Ingenuity (IPA) software. **Fig. S4** Prenatal exposure to E_3_ has no effect on learning or memory behavior. Eight weeks-old pregnant female CD-1 mice were treated with vehicle DMSO (CT) or E_3_. At 6 months after birth, the offspring were subjected to a water maze test and novel object recognition (NOR) task. **A)** During the day 2-5 of invisible platform tests, the E_3_ treated and CT mice exhibited a similar latency to escape onto the hidden platform. **B-D**) In the probe trial on the 6^th^ day, the E_3_-treated offspring traveled into the third quadrant, where the hidden platform was previously placed. The **B)** passing time, **C)** time spent in the area and **D)** path length are similar between E_3_ and CT offspring. **E)** shows the time spent on two objects (initial object labeled A) at day 1, versus repeated object A and a new object B at day 3. The mice spent a longer amount of time on object B (new object) than object A (old object) at day 3. There was no difference in the time for either object A or B between CT and E_3_. Error bars: mean + SEM of the results, P>0.05 (Student t-test), N=24 for each CT and E_3_. **Fig. S5** Pathway analysis of the anxiety related genes with altered expression in the E_3_-treated offspring. A) Pathway analysis of the anxiety related genes with altered expression in the Male and Female offspring cortex and verified by RT-PCR (Fig.5B-E), B) Pathway analysis of the anxiety related genes with altered expression in the female offspring hippocampus and verified by RT-PCR (Fig.5 A). **Fig. S6**. Prenatal exposure to E_3_ alters global gene expression in the adult offspring brains. Eight weeks-old pregnant female CD-1 mice were treated with vehicle DMSO (CT) or E_3_. 6 months after birth, the gene expression in the offspring brains was profiled by a whole genome mRNA microarray. The altered genes are grouped according to their biological functions in **A)** female hippocampus, **C)** male hippocampus, **E)** male cortex and **G**) female cortex. The altered genes that are related to the ERβ (also known as ESR2) signaling are shown in **B)** female hippocampus, , **F)** male cortex, **H)** female cortex, **D)** related to the ERα (also known as ESR1) signaling in male hippocampus. The data are analyzed using the Ingenuity (IPA) software.

**Additional file 2: Table S1.** PCR primers. **Table S2.** The genes with altered expression patterns in the female offspring uteri after prenatal exposure to estriol (E_3_ v.s CT). **Table S3. A** The hypomethylated genes in the female offspring uteri after prenatal exposure to estriol (E_3_ vs CT). **B** The hypermethylated genes in the female offspring uteri after prenatal exposure to estriol (E_3_ vs CT). **Table S4**. The genes with altered E_2_ responsiveness in the female offspring uteri after prenatal exposure to estriol (E_3_-E_2_ vs CT-E_2_). **Table S5.** The potential binding proteins with Flag-ERα, Flag-ERβ in Ishikawa cells by Multi-Dimensional Protein Identification Technology (MudPIT). **Table S6.** The genes that are bound by the ERα-SUZ12 and ERβ-SUZ12 protein complex in Ishikawa cells. The Re-ChIP results show that E_3_ treatment influences binding of ER-SUZ12 complex to their target genes in Ishikawa cells*.* **Table S7.** The genes with altered expression patterns (≥ 1.5 or ≤ -1.5-fold change) in the female offspring cortex and hippocampus after prenatal exposure to estriol (E_3_ vs CT). **Table S8.** The top 3000 CpG sites in the female offspring prefrontal cortex after prenatal exposure to estriol (E_3_ vs CT). **Table S9** The genes that are bound by the ERα-DNMT1, ERβ-DNMT1, ERα-SUZ12 and ERβ-SUZ12 protein complex in SY5Y cells. By a Re-ChIP assay, we identified 2391 DNA promotor sites that were bound by both ERα and DNMT1 simultaneously, 53 sites were bound by both ERβ and DNMT1; 253 sites were bound by both ERα and SUZ12, and 260 sites were bound by both ERβ and SUZ12, after E_3_ treatment. **Table S10**. A**)** The top 2000 hypermethylated differential CpG sites in the female offspring hippocampus after prenatal exposure to estriol (E_3_ vs CT). **B)** The top 2000 hypomethylated differential CpG sites in the female offspring hippocampus after prenatal exposure to estriol (E_3_ vs CT).

**Additional file 3.** Uncropped blots.

## Data Availability

All data generated or analyzed during this study are included in this published article, supplementary information files, and publicly available repositories. mRNA Microarray, DNA methylation array, Methylation mini-Sequencing, and Re-chip-Sequencing data are available in the GEO 1repository (https://www.ncbi.nlm.nih.gov/geo/query/acc.cgi?acc=GSE199214).

## References

[1] Head KA (1998). Estriol: safety and efficacy. Altern Med Rev.

[2] Longcope C (1984). Estriol production and metabolism in normal women. J Steroid Biochem.

[3] Flood C, Pratt JH, Longcope C (1976). The metabolic clearance and blood production rates of estriol in normal, non-pregnant women. J Clin Endocrinol Metab.

[4] Kuijper EA, Ket JC, Caanen MR, Lambalk CB (2013). Reproductive hormone concentrations in pregnancy and neonates: a systematic review. Reprod Biomed Online.

[5] Mesiano S. Chapter 11 - Endocrinology of Human Pregnancy and Fetal-Placental Neuroendocrine Development. Editor(s): Jerome F. Strauss, Robert L. Barbieri, Yen and Jaffe's Reproductive Endocrinology (Eighth Edition), Elsevier. 2019:256-284.e9, ISBN 9780323479127. 10.1016/B978-0-323-47912-7.00011-1.

[6] Albrecht ED, Pepe GJ (2010). Estrogen regulation of placental angiogenesis and fetal ovarian development during primate pregnancy. Int J Dev Biol.

[7] Laredo SA, Villalon Landeros R, Trainor BC (2014). Rapid effects of estrogens on behavior: environmental modulation and molecular mechanisms. Front Neuroendocrinol.

[8] Rich RL, Hoth LR, Geoghegan KF, Brown TA, LeMotte PK, Simons SP, Hensley P, Myszka DG (2002). Kinetic analysis of estrogen receptor/ligand interactions. Proc Natl Acad Sci U S A.

[9] Rettberg JR, Yao J, Brinton RD (2014). Estrogen: a master regulator of bioenergetic systems in the brain and body. Front Neuroendocrinol.

[10] Hong H, Branham WS, Ng HW, Moland CL, Dial SL, Fang H, Perkins R, Sheehan D, Tong W (2015). Human sex hormone-binding globulin binding affinities of 125 structurally diverse chemicals and comparison with their binding to androgen receptor, estrogen receptor, and alpha-fetoprotein. Toxicol Sci.

[11] Bromer JG, Wu J, Zhou Y, Taylor HS (2009). Hypermethylation of homeobox A10 by in utero diethylstilbestrol exposure: an epigenetic mechanism for altered developmental programming. Endocrinology.

[12] Bromer JG, Zhou Y, Taylor MB, Doherty L, Taylor HS (2010). Bisphenol-A exposure in utero leads to epigenetic alterations in the developmental programming of uterine estrogen response. FASEB J.

[13] Akbas GE, Song J, Taylor HS (2004). A HOXA10 estrogen response element (ERE) is differentially regulated by 17 beta-estradiol and diethylstilbestrol (DES). J Mol Biol.

[14] Doherty LF, Bromer JG, Zhou Y, Aldad TS, Taylor HS (2010). In utero exposure to diethylstilbestrol (DES) or bisphenol-A (BPA) increases EZH2 expression in the mammary gland: an epigenetic mechanism linking endocrine disruptors to breast cancer. Horm Cancer.

[15] Shi JF, Li XJ, Si XX, Li AD, Ding HJ, Han X, Sun YJ (2012). ERalpha positively regulated DNMT1 expression by binding to the gene promoter region in human breast cancer MCF-7 cells. Biochem Biophys Res Commun.

[16] Cui M, Wen Z, Yang Z, Chen J, Wang F (2009). Estrogen regulates DNA methyltransferase 3B expression in Ishikawa endometrial adenocarcinoma cells. Mol Biol Rep.

[17] Russell JK, Jones CK, Newhouse PA (2019). The role of estrogen in brain and cognitive aging. Neurotherapeutics.

[18] Vegeto E, Villa A, Della Torre S, Crippa V, Rusmini P, Cristofani R, et al. The role of sex and sex hormones in neurodegenerative diseases. Endocr Rev. 2020;41(2):273-319. 10.1210/endrev/bnz005 PMC7156855 31544208

[19] Santagati S, Melcangi RC, Celotti F, Martini L, Maggi A (1994). Estrogen receptor is expressed in different types of glial cells in culture. J Neurochem.

[20] Osterlund MK, Hurd YL (2001). Estrogen receptors in the human forebrain and the relation to neuropsychiatric disorders. Prog Neurobiol.

[21] Wang S, Che T, Levit A, Shoichet BK, Wacker D, Roth BL (2018). Structure of the D2 dopamine receptor bound to the atypical antipsychotic drug risperidone. Nature.

[22] Perkins MS, Louw-du Toit R, Africander D (2017). A comparative characterization of estrogens used in hormone therapy via estrogen receptor (ER)-alpha and -beta. J Steroid Biochem Mol Biol.

[23] Kuiper GG, Carlsson B, Grandien K, Enmark E, Haggblad J, Nilsson S, Gustafsson JA (1997). Comparison of the ligand binding specificity and transcript tissue distribution of estrogen receptors alpha and beta. Endocrinology.

[24] Sasson S, Notides AC (1984). The estriol-induced inhibition of the estrogen receptor's positive cooperativity. J Steroid Biochem.

[25] Kaneko K, Choudhuri S. Chapter 52 - epigenetics in reproduction and development. In: Gupta RC, editor. Reproductive and Developmental Toxicology. 2nd ed: Academic Press; 2017. p. 1005–21. 10.1016/B978-0-12-804239-7.00052-4

[26] Wu Y, Strawn E, Basir Z, Halverson G, Guo SW (2007). Aberrant expression of deoxyribonucleic acid methyltransferases DNMT1, DNMT3A, and DNMT3B in women with endometriosis. Fertil Steril.

[27] Perillo B, Ombra MN, Bertoni A, Cuozzo C, Sacchetti S, Sasso A, Chiariotti L, Malorni A, Abbondanza C, Avvedimento EV (2008). DNA oxidation as triggered by H3K9me2 demethylation drives estrogen-induced gene expression. Science.

[28] Shi YJ, Matson C, Lan F, Iwase S, Baba T, Shi Y (2005). Regulation of LSD1 histone demethylase activity by its associated factors. Mol Cell.

[29] Wang J, Hevi S, Kurash JK, Lei H, Gay F, Bajko J, Su H, Sun W, Chang H, Xu G (2009). The lysine demethylase LSD1 (KDM1) is required for maintenance of global DNA methylation. Nat Genet.

[30] Frick KM (2015). Molecular mechanisms underlying the memory-enhancing effects of estradiol. Horm Behav.

[31] Levin ER (2005). Integration of the extranuclear and nuclear actions of estrogen. Mol Endocrinol.

[32] Revankar CM, Cimino DF, Sklar LA, Arterburn JB, Prossnitz ER (2005). A transmembrane intracellular estrogen receptor mediates rapid cell signaling. Science.

[33] Vasudevan N, Pfaff DW (2008). Non-genomic actions of estrogens and their interaction with genomic actions in the brain. Front Neuroendocrinol.

[34] Nayak V, Xu C, Min J (2011). Composition, recruitment and regulation of the PRC2 complex. Nucleus.

[35] Smith CC, Taylor HS (2007). Xenoestrogen exposure imprints expression of genes (Hoxa10) required for normal uterine development. FASEB J.

[36] Jorgensen EM, Alderman MH, Taylor HS (2016). Preferential epigenetic programming of estrogen response after in utero xenoestrogen (bisphenol-A) exposure. FASEB J.

[37] Bruning JB, Parent AA, Gil G, Zhao M, Nowak J, Pace MC, Smith CL, Afonine PV, Adams PD, Katzenellenbogen JA (2010). Coupling of receptor conformation and ligand orientation determine graded activity. Nat Chem Biol.

[38] Mueller SO, Hall JM, Swope DL, Pedersen LC, Korach KS (2003). Molecular determinants of the stereoselectivity of agonist activity of estrogen receptors (ER) alpha and beta. J Biol Chem.

[39] Hansen JC, Gorski J (1986). Conformational transitions of the estrogen receptor monomer. Effects of estrogens, antiestrogen, and temperature. J Biol Chem.

[40] Shiau AK, Barstad D, Loria PM, Cheng L, Kushner PJ, Agard DA, Greene GL (1998). The structural basis of estrogen receptor/coactivator recognition and the antagonism of this interaction by tamoxifen. Cell.

[41] Paige LA, Christensen DJ, Gron H, Norris JD, Gottlin EB, Padilla KM, Chang CY, Ballas LM, Hamilton PT, McDonnell DP (1999). Estrogen receptor (ER) modulators each induce distinct conformational changes in ER alpha and ER beta. Proc Natl Acad Sci U S A.

[42] Wu YL, Yang X, Ren Z, McDonnell DP, Norris JD, Willson TM, Greene GL (2005). Structural basis for an unexpected mode of SERM-mediated ER antagonism. Mol Cell.

[43] Lupien M, Jeyakumar M, Hebert E, Hilmi K, Cotnoir-White D, Loch C, Auger A, Dayan G, Pinard GA, Wurtz JM (2007). Raloxifene and ICI182,780 increase estrogen receptor-alpha association with a nuclear compartment via overlapping sets of hydrophobic amino acids in activation function 2 helix 12. Mol Endocrinol.

[44] Vrtacnik P, Ostanek B, Mencej-Bedrac S, Marc J (2014). The many faces of estrogen signaling. Biochem Med (Zagreb).

[45] Liu ZW, Faraguna U, Cirelli C, Tononi G, Gao XB (2010). Direct evidence for wake-related increases and sleep-related decreases in synaptic strength in rodent cortex. J Neurosci.

[46] Arrant AE, Schramm-Sapyta NL, Kuhn CM (2013). Use of the light/dark test for anxiety in adult and adolescent male rats. Behav Brain Res.

[47] Takao K, Miyakawa T (2006). Light/dark transition test for mice. J Vis Exp.

[48] Lueptow LM. Novel Object Recognition Test for the Investigation of Learning and Memory in Mice. J Vis Exp. 2017;126:55718. 10.3791/55718 PMC5614391 28892027

[49] Bromley-Brits K, Deng Y, Song W. Morris water maze test for learning and memory deficits in Alzheimer's disease model mice. J Vis Exp. 2011;53:2920. 10.3791/2920 PMC3347885 21808223

[50] Liu ZW, Gao XB (2007). Adenosine inhibits activity of hypocretin/orexin neurons by the A1 receptor in the lateral hypothalamus: a possible sleep-promoting effect. J Neurophysiol.

[51] Liu ZW, Gan G, Suyama S, Gao XB (2011). Intracellular energy status regulates activity in hypocretin/orexin neurones: a link between energy and behavioural states. J Physiol.

[52] Ruan HB, Han X, Li MD, Singh JP, Qian K, Azarhoush S, Zhao L, Bennett AM, Samuel VT, Wu J (2012). O-GlcNAc transferase/host cell factor C1 complex regulates gluconeogenesis by modulating PGC-1alpha stability. Cell Metab.

