# Supplementary figures and images for: The steroid hormone estriol (E3) regulates epigenetic programming of fetal mouse brain and reproductive tract
Source: BMC Biol. 2022 May 2;20:93. doi: 10.1186/s12915-022-01293-4 (PMC9059368; doi:10.1186/s12915-022-01293-4)

## A

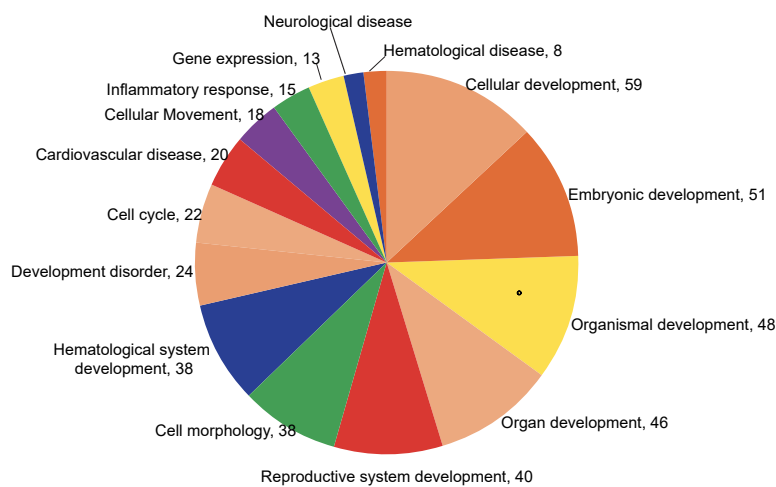

B

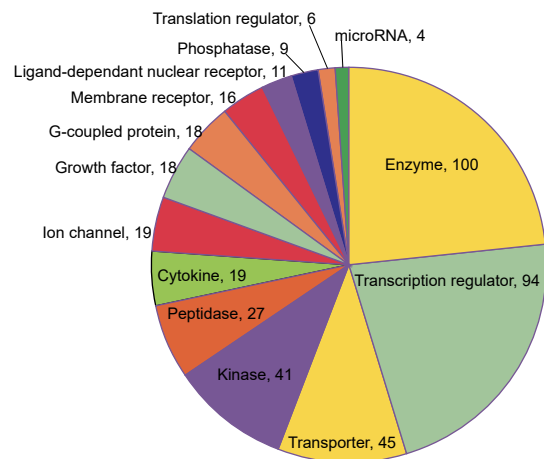

C

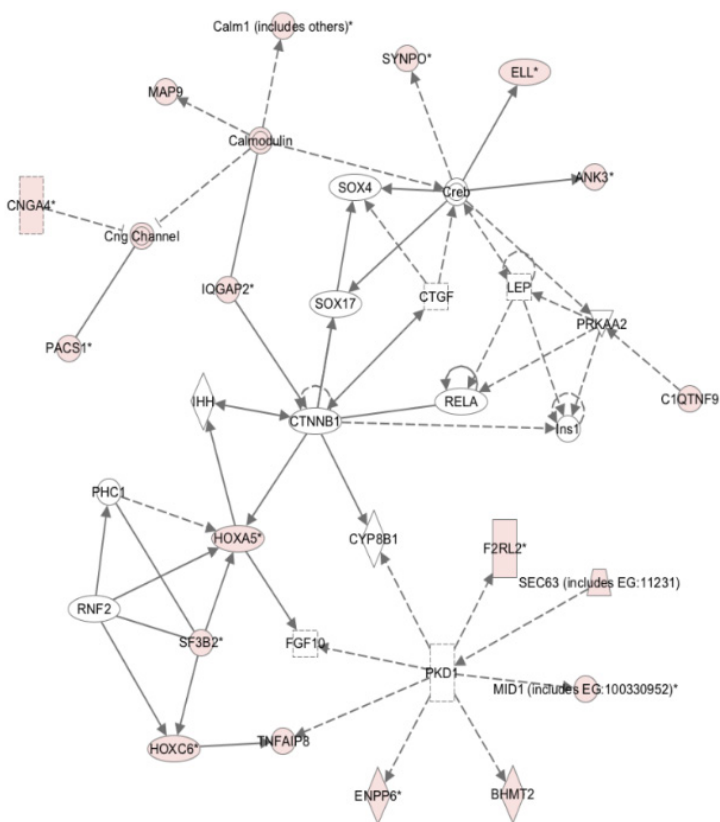

D

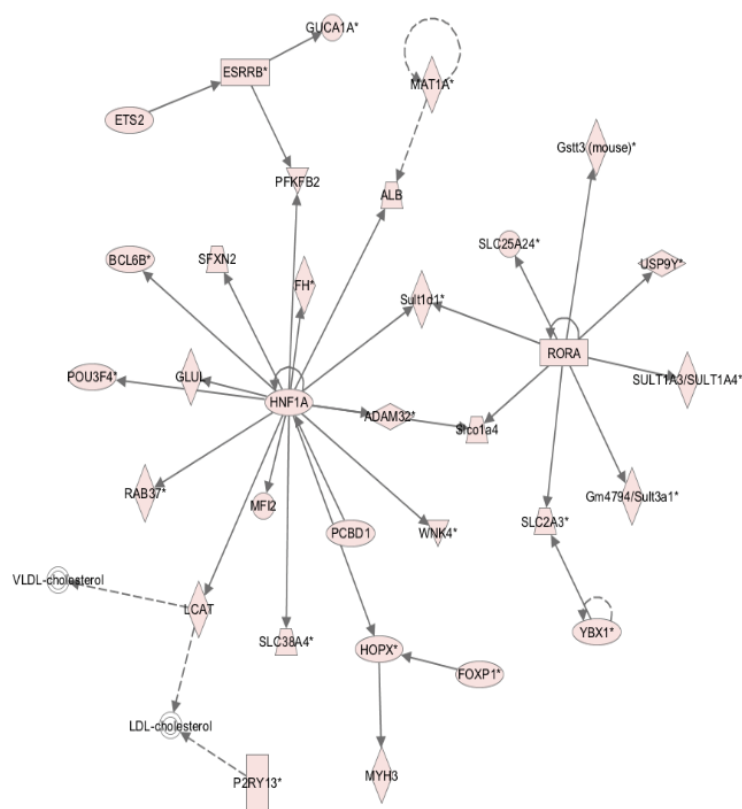

E

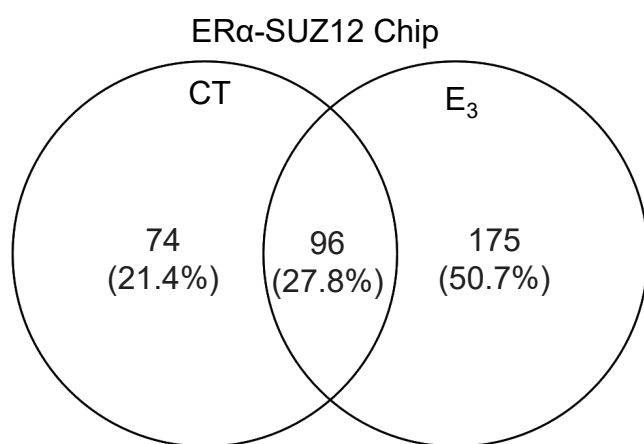

F

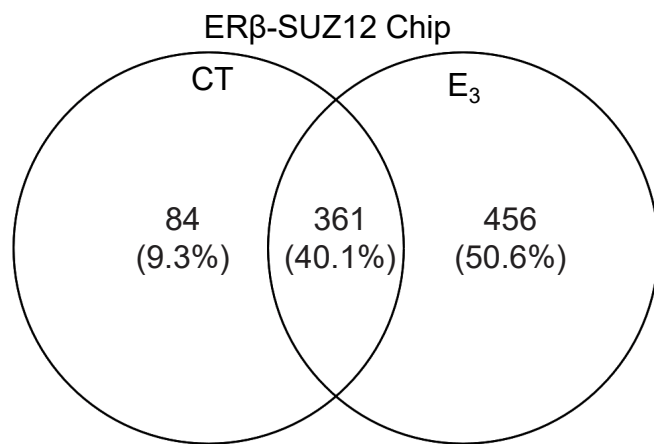

Supplement: Supplementary file 1 — Additional file 1: Fig. S1 Prenatal exposure of estriol (E3) alters the global gene expression profile of the female offspring uteri. A) Grouping of altered genes in E3-treated animals compared to vehicle-treated groups according to biological functions using the Ingenuity (IPA) software. B) Pathway analyses using Ingenuity (IPA) software. Fig. S2 A, B, C, D) Prenatal exposure of E3 results in significant hypomethylation of over 2000 genes in the female offspring uteri. Grouping of altered genes in E3-treated compared to vehicle-treated groups according to A) biological functions and B) molecular functions using Ingenuity (IPA) software. C-D) Pathway analyses using Ingenuity (IPA) software. E, F) E3 treatment increases the numbers of ER-SUZ12-bound genes. Ishikawa cells were treated with/without E3, and ChIP (chromatin immunoprecipitation) was performed using an E) anti-estrogen receptor α (ERα) or F) ERβ antibody (First ChIP) and then with an anti-SUZ12 antibody (Re-ChIP). The bound DNA was then identified by sequencing. The Venn diagrams show the numbers of genes bound by the ER-SUZ12 complex with/without E3 treatment. Fig. S3) Prenatal exposure to E3 results in significant hypermethylation of over 2500 genes in the female offspring uteri. Grouping of altered genes in E3-treated compared to vehicle-treated groups according to A) biological functions and B) molecular functions using the Ingenuity (IPA) software. C-D) Pathway analyses using Ingenuity (IPA) software. Fig. S4 Prenatal exposure to E3 has no effect on learning or memory behavior. Eight weeks-old pregnant female CD-1 mice were treated with vehicle DMSO (CT) or E3. At 6 months after birth, the offspring were subjected to a water maze test and novel object recognition (NOR) task. A) During the day 2-5 of invisible platform tests, the E3 treated and CT mice exhibited a similar latency to escape onto the hidden platform. B-D) In the probe trial on the 6th day, the E3-treated offspring traveled into the third [file 12915_2022_1293_MOESM1_ESM.zip › ExtFig2 (2).pdf]

# Supplemental Figure 3

A

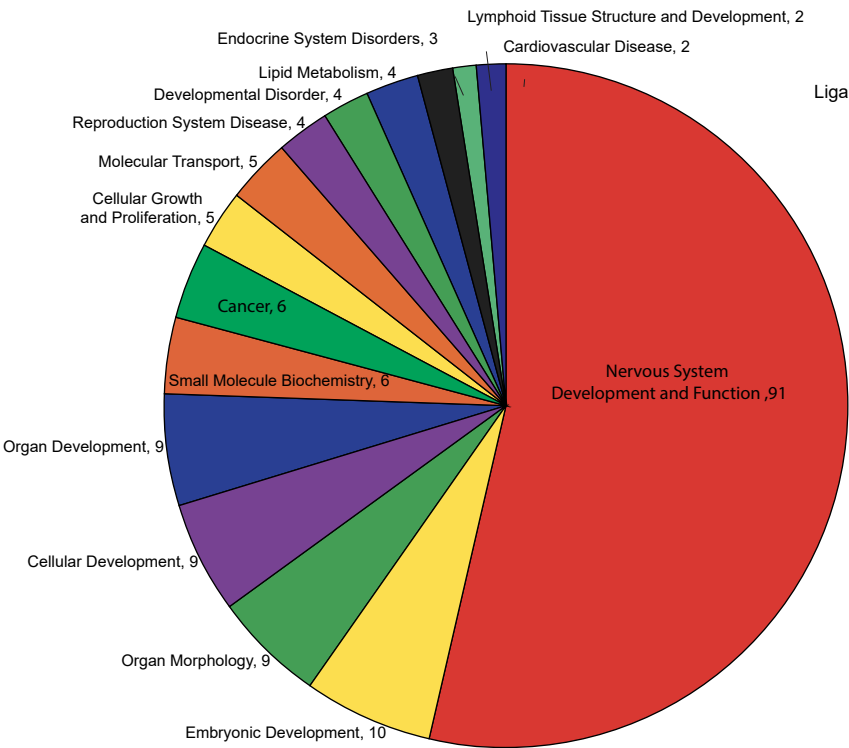

B

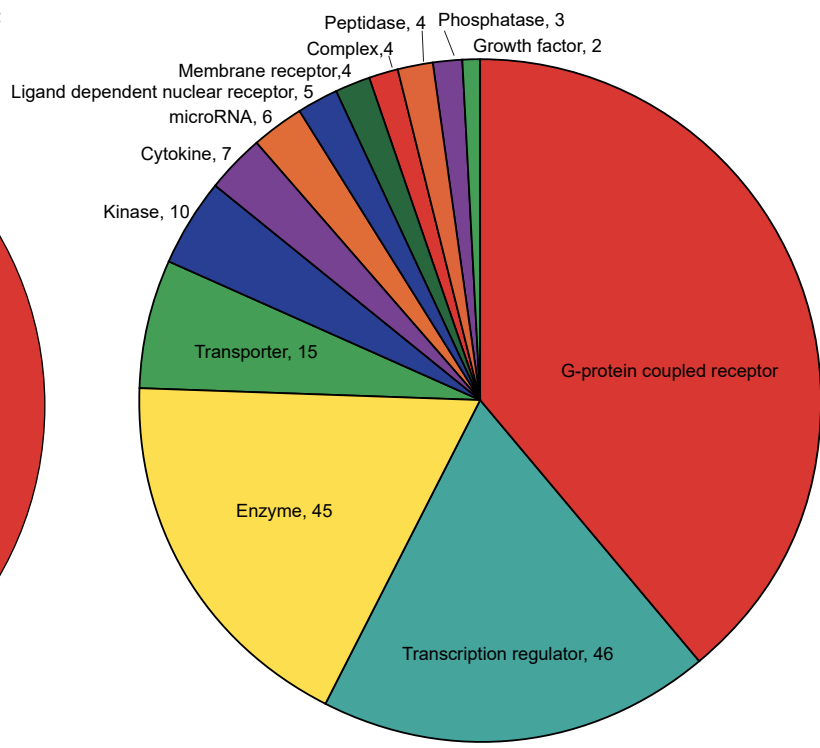

C

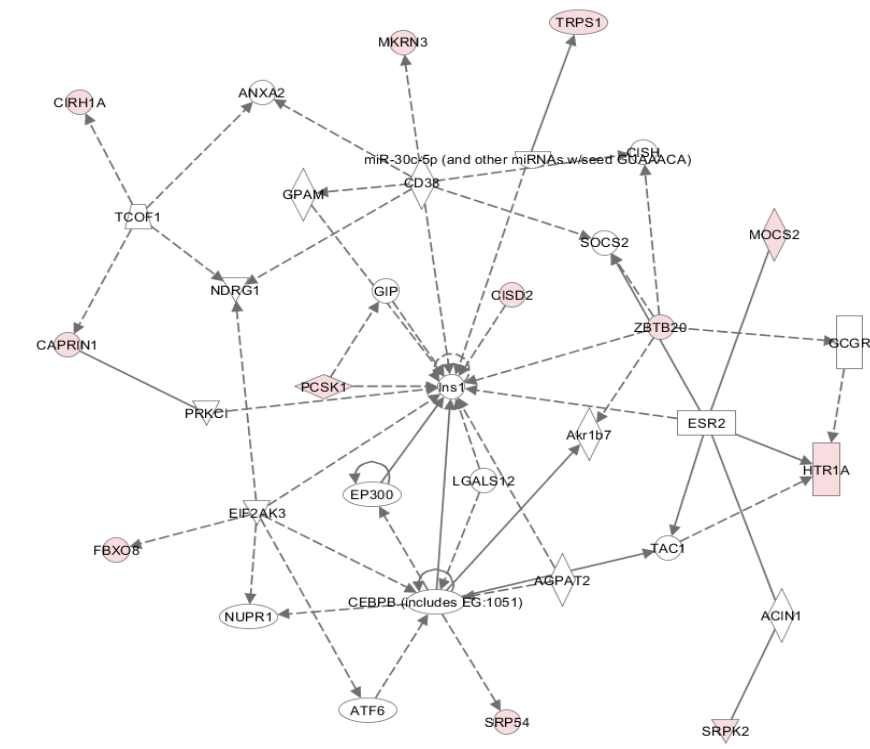

D

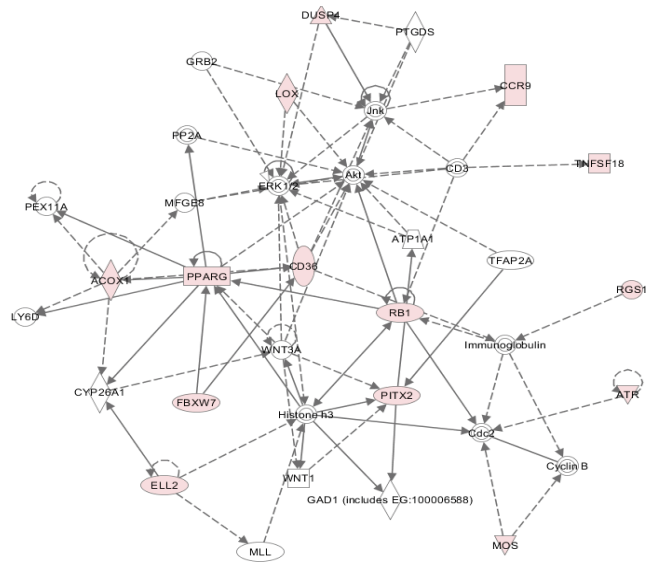

Supplement: Supplementary file 1 — Additional file 1: Fig. S1 Prenatal exposure of estriol (E3) alters the global gene expression profile of the female offspring uteri. A) Grouping of altered genes in E3-treated animals compared to vehicle-treated groups according to biological functions using the Ingenuity (IPA) software. B) Pathway analyses using Ingenuity (IPA) software. Fig. S2 A, B, C, D) Prenatal exposure of E3 results in significant hypomethylation of over 2000 genes in the female offspring uteri. Grouping of altered genes in E3-treated compared to vehicle-treated groups according to A) biological functions and B) molecular functions using Ingenuity (IPA) software. C-D) Pathway analyses using Ingenuity (IPA) software. E, F) E3 treatment increases the numbers of ER-SUZ12-bound genes. Ishikawa cells were treated with/without E3, and ChIP (chromatin immunoprecipitation) was performed using an E) anti-estrogen receptor α (ERα) or F) ERβ antibody (First ChIP) and then with an anti-SUZ12 antibody (Re-ChIP). The bound DNA was then identified by sequencing. The Venn diagrams show the numbers of genes bound by the ER-SUZ12 complex with/without E3 treatment. Fig. S3) Prenatal exposure to E3 results in significant hypermethylation of over 2500 genes in the female offspring uteri. Grouping of altered genes in E3-treated compared to vehicle-treated groups according to A) biological functions and B) molecular functions using the Ingenuity (IPA) software. C-D) Pathway analyses using Ingenuity (IPA) software. Fig. S4 Prenatal exposure to E3 has no effect on learning or memory behavior. Eight weeks-old pregnant female CD-1 mice were treated with vehicle DMSO (CT) or E3. At 6 months after birth, the offspring were subjected to a water maze test and novel object recognition (NOR) task. A) During the day 2-5 of invisible platform tests, the E3 treated and CT mice exhibited a similar latency to escape onto the hidden platform. B-D) In the probe trial on the 6th day, the E3-treated offspring traveled into the third [file 12915_2022_1293_MOESM1_ESM.zip › ExtFig3 (2).pdf]

# Supplemental Figure 4

A

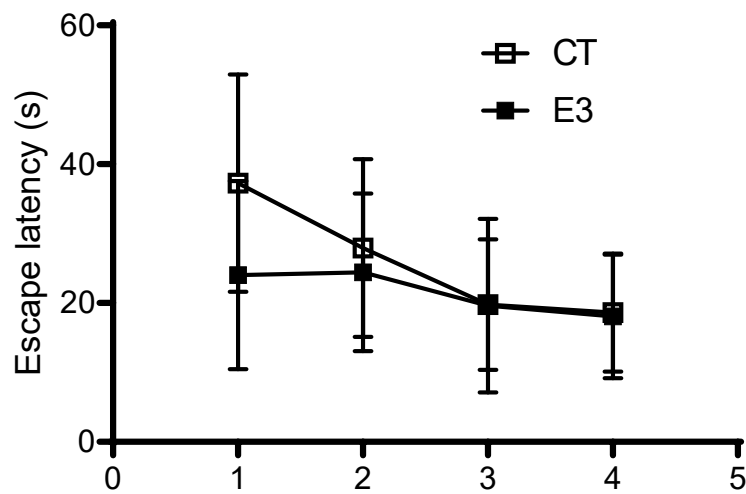

B

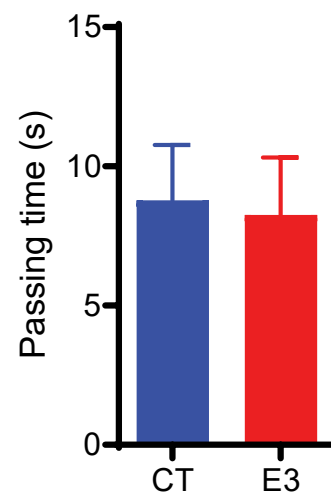

C

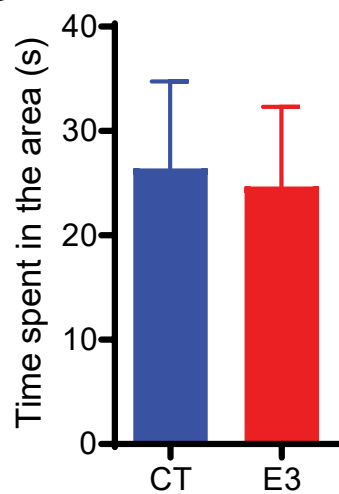

D

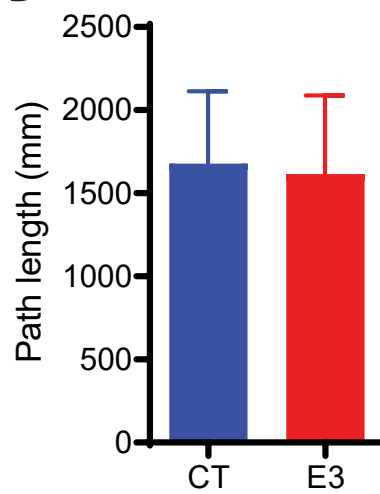

E

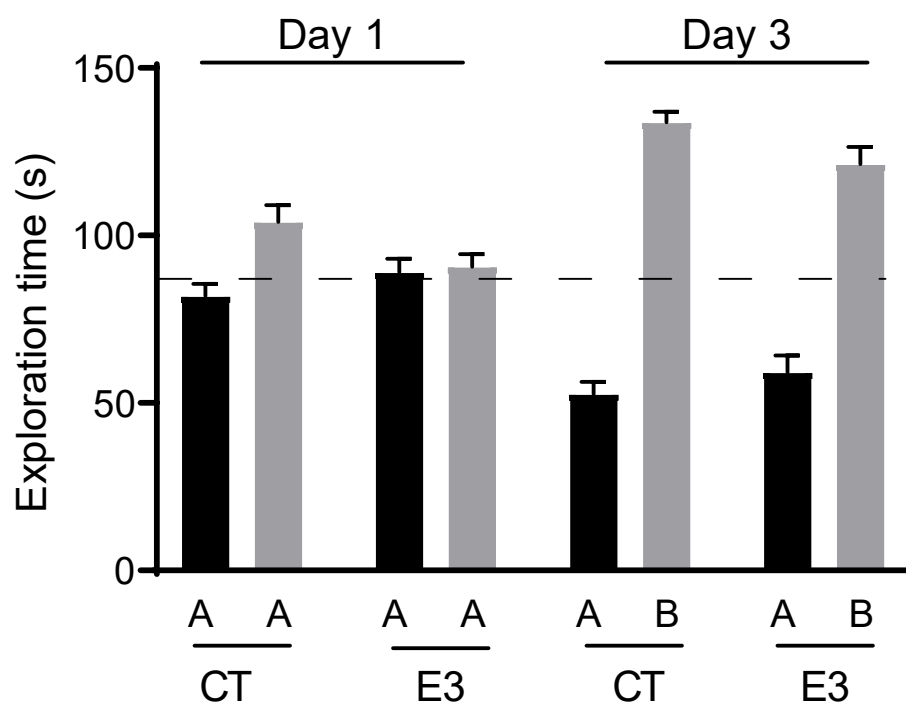

Supplement: Supplementary file 1 — Additional file 1: Fig. S1 Prenatal exposure of estriol (E3) alters the global gene expression profile of the female offspring uteri. A) Grouping of altered genes in E3-treated animals compared to vehicle-treated groups according to biological functions using the Ingenuity (IPA) software. B) Pathway analyses using Ingenuity (IPA) software. Fig. S2 A, B, C, D) Prenatal exposure of E3 results in significant hypomethylation of over 2000 genes in the female offspring uteri. Grouping of altered genes in E3-treated compared to vehicle-treated groups according to A) biological functions and B) molecular functions using Ingenuity (IPA) software. C-D) Pathway analyses using Ingenuity (IPA) software. E, F) E3 treatment increases the numbers of ER-SUZ12-bound genes. Ishikawa cells were treated with/without E3, and ChIP (chromatin immunoprecipitation) was performed using an E) anti-estrogen receptor α (ERα) or F) ERβ antibody (First ChIP) and then with an anti-SUZ12 antibody (Re-ChIP). The bound DNA was then identified by sequencing. The Venn diagrams show the numbers of genes bound by the ER-SUZ12 complex with/without E3 treatment. Fig. S3) Prenatal exposure to E3 results in significant hypermethylation of over 2500 genes in the female offspring uteri. Grouping of altered genes in E3-treated compared to vehicle-treated groups according to A) biological functions and B) molecular functions using the Ingenuity (IPA) software. C-D) Pathway analyses using Ingenuity (IPA) software. Fig. S4 Prenatal exposure to E3 has no effect on learning or memory behavior. Eight weeks-old pregnant female CD-1 mice were treated with vehicle DMSO (CT) or E3. At 6 months after birth, the offspring were subjected to a water maze test and novel object recognition (NOR) task. A) During the day 2-5 of invisible platform tests, the E3 treated and CT mice exhibited a similar latency to escape onto the hidden platform. B-D) In the probe trial on the 6th day, the E3-treated offspring traveled into the third [file 12915_2022_1293_MOESM1_ESM.zip › ExtFig4 (2).pdf]

# A Supplemental Figure 5

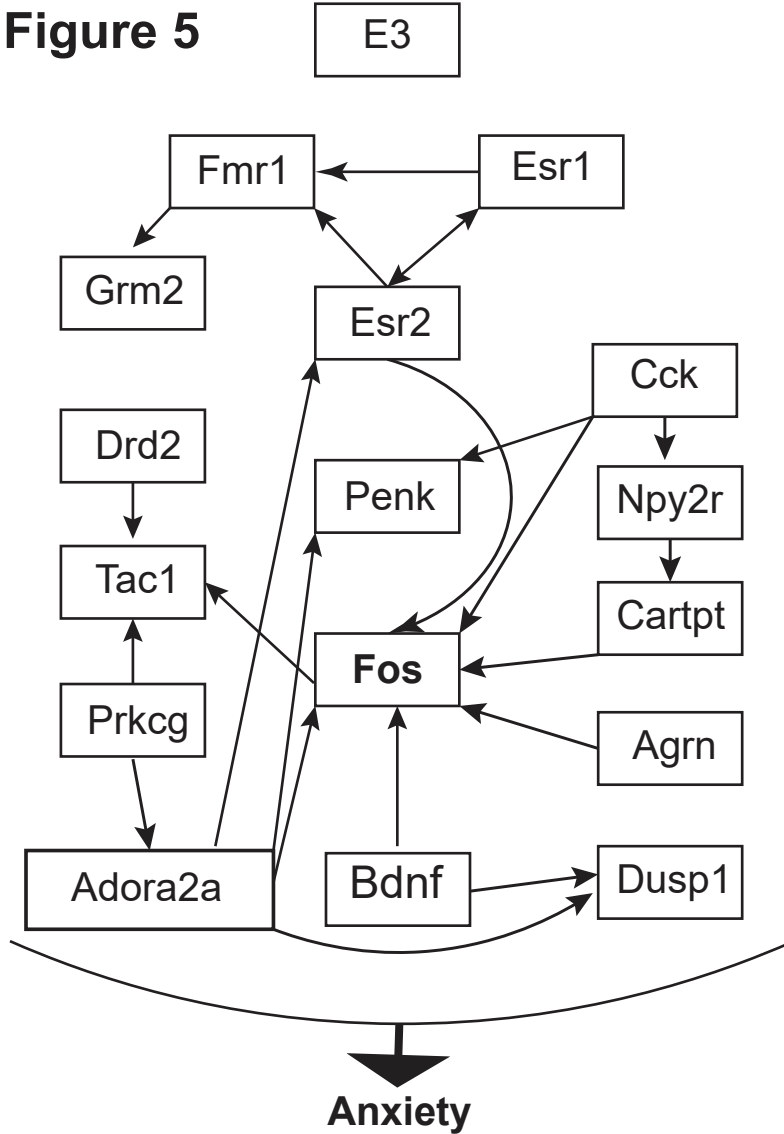

# B

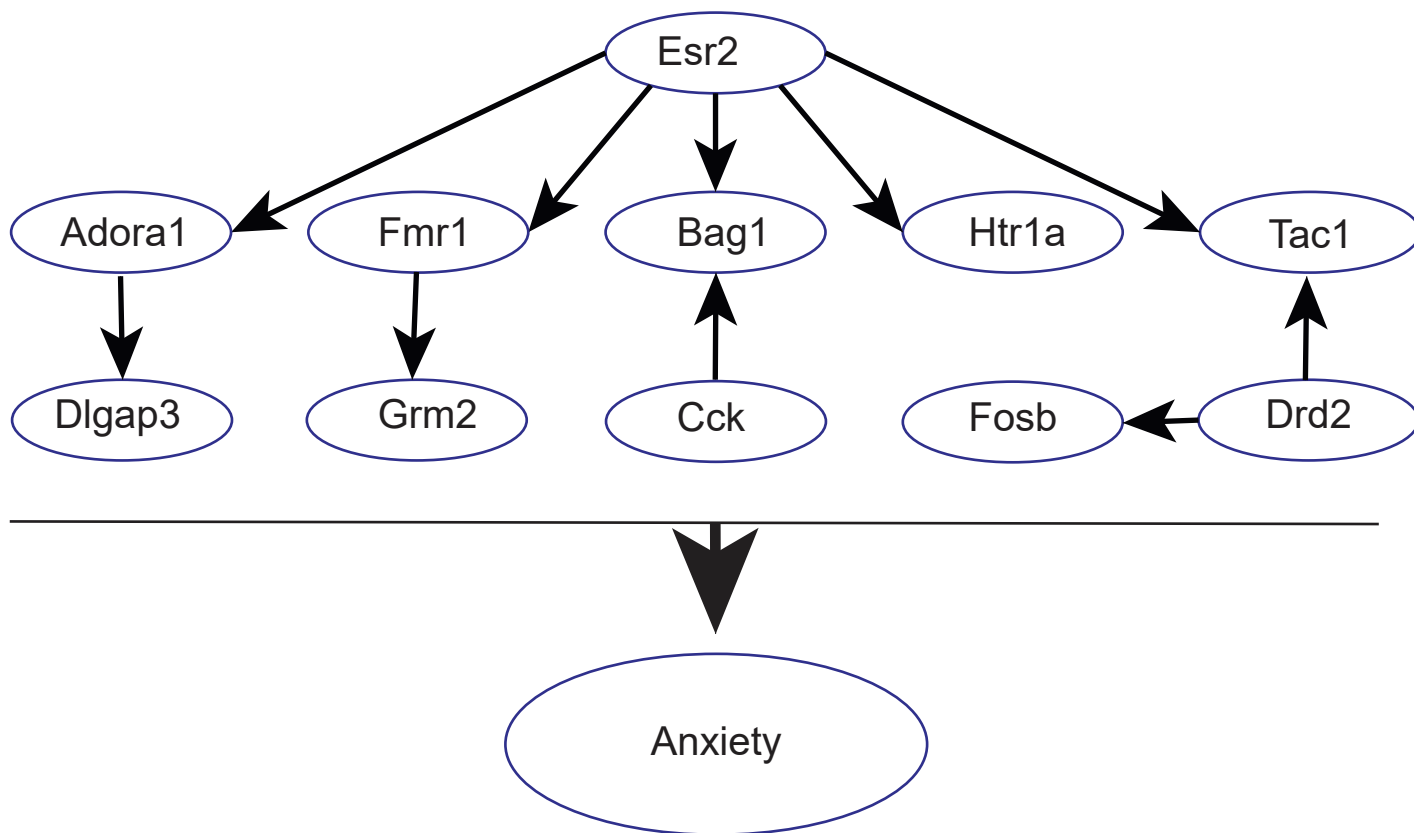

Supplement: Supplementary file 1 — Additional file 1: Fig. S1 Prenatal exposure of estriol (E3) alters the global gene expression profile of the female offspring uteri. A) Grouping of altered genes in E3-treated animals compared to vehicle-treated groups according to biological functions using the Ingenuity (IPA) software. B) Pathway analyses using Ingenuity (IPA) software. Fig. S2 A, B, C, D) Prenatal exposure of E3 results in significant hypomethylation of over 2000 genes in the female offspring uteri. Grouping of altered genes in E3-treated compared to vehicle-treated groups according to A) biological functions and B) molecular functions using Ingenuity (IPA) software. C-D) Pathway analyses using Ingenuity (IPA) software. E, F) E3 treatment increases the numbers of ER-SUZ12-bound genes. Ishikawa cells were treated with/without E3, and ChIP (chromatin immunoprecipitation) was performed using an E) anti-estrogen receptor α (ERα) or F) ERβ antibody (First ChIP) and then with an anti-SUZ12 antibody (Re-ChIP). The bound DNA was then identified by sequencing. The Venn diagrams show the numbers of genes bound by the ER-SUZ12 complex with/without E3 treatment. Fig. S3) Prenatal exposure to E3 results in significant hypermethylation of over 2500 genes in the female offspring uteri. Grouping of altered genes in E3-treated compared to vehicle-treated groups according to A) biological functions and B) molecular functions using the Ingenuity (IPA) software. C-D) Pathway analyses using Ingenuity (IPA) software. Fig. S4 Prenatal exposure to E3 has no effect on learning or memory behavior. Eight weeks-old pregnant female CD-1 mice were treated with vehicle DMSO (CT) or E3. At 6 months after birth, the offspring were subjected to a water maze test and novel object recognition (NOR) task. A) During the day 2-5 of invisible platform tests, the E3 treated and CT mice exhibited a similar latency to escape onto the hidden platform. B-D) In the probe trial on the 6th day, the E3-treated offspring traveled into the third [file 12915_2022_1293_MOESM1_ESM.zip › ExtFig5 (2).pdf]

A

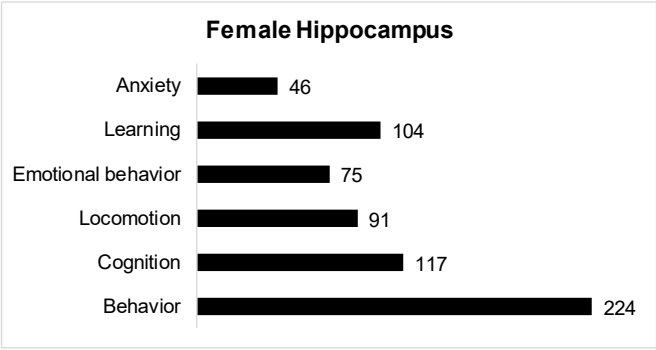

B

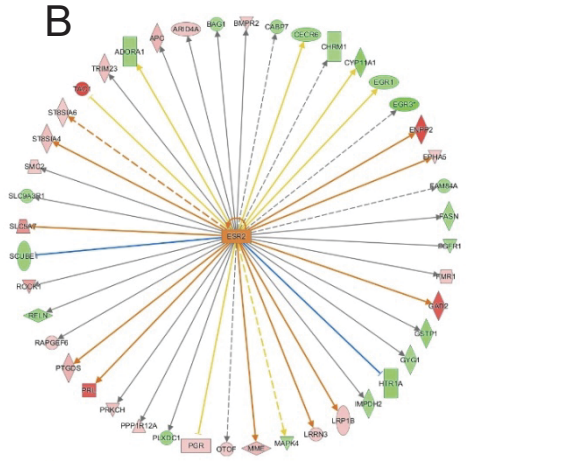

C

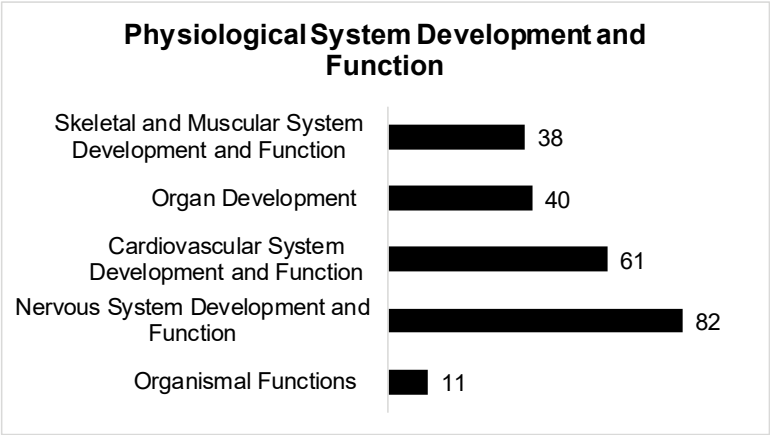

D

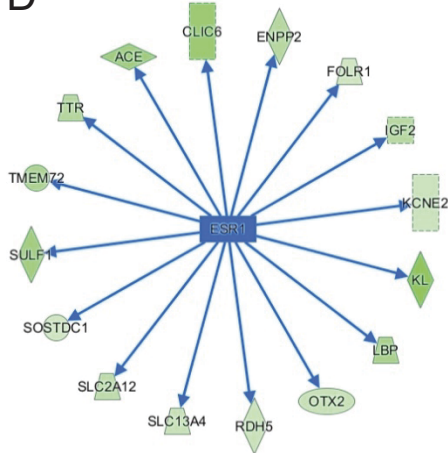

E

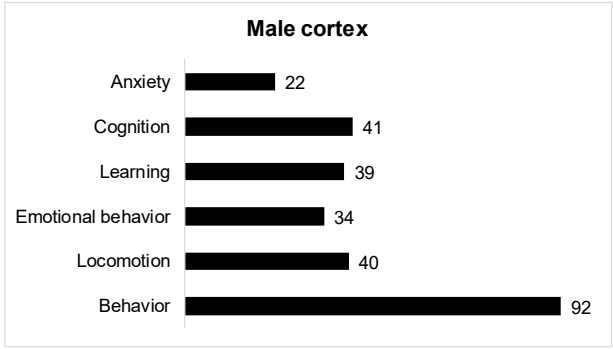

F

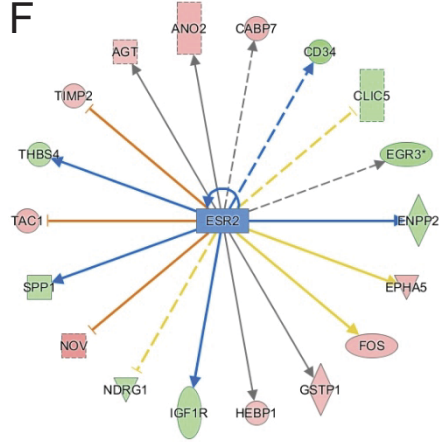

G

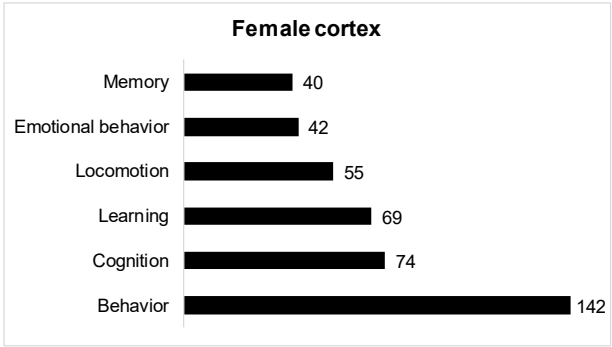

H

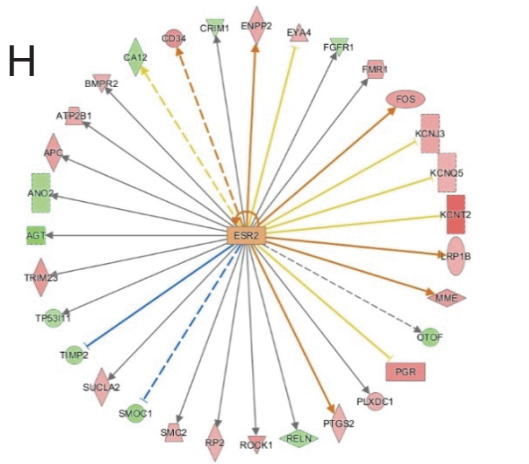

Supplement: Supplementary file 1 — Additional file 1: Fig. S1 Prenatal exposure of estriol (E3) alters the global gene expression profile of the female offspring uteri. A) Grouping of altered genes in E3-treated animals compared to vehicle-treated groups according to biological functions using the Ingenuity (IPA) software. B) Pathway analyses using Ingenuity (IPA) software. Fig. S2 A, B, C, D) Prenatal exposure of E3 results in significant hypomethylation of over 2000 genes in the female offspring uteri. Grouping of altered genes in E3-treated compared to vehicle-treated groups according to A) biological functions and B) molecular functions using Ingenuity (IPA) software. C-D) Pathway analyses using Ingenuity (IPA) software. E, F) E3 treatment increases the numbers of ER-SUZ12-bound genes. Ishikawa cells were treated with/without E3, and ChIP (chromatin immunoprecipitation) was performed using an E) anti-estrogen receptor α (ERα) or F) ERβ antibody (First ChIP) and then with an anti-SUZ12 antibody (Re-ChIP). The bound DNA was then identified by sequencing. The Venn diagrams show the numbers of genes bound by the ER-SUZ12 complex with/without E3 treatment. Fig. S3) Prenatal exposure to E3 results in significant hypermethylation of over 2500 genes in the female offspring uteri. Grouping of altered genes in E3-treated compared to vehicle-treated groups according to A) biological functions and B) molecular functions using the Ingenuity (IPA) software. C-D) Pathway analyses using Ingenuity (IPA) software. Fig. S4 Prenatal exposure to E3 has no effect on learning or memory behavior. Eight weeks-old pregnant female CD-1 mice were treated with vehicle DMSO (CT) or E3. At 6 months after birth, the offspring were subjected to a water maze test and novel object recognition (NOR) task. A) During the day 2-5 of invisible platform tests, the E3 treated and CT mice exhibited a similar latency to escape onto the hidden platform. B-D) In the probe trial on the 6th day, the E3-treated offspring traveled into the third [file 12915_2022_1293_MOESM1_ESM.zip › ExtFig6 (2).pdf]
