# Supplementary material for: The steroid hormone estriol (E3) regulates epigenetic programming of fetal mouse brain and reproductive tract
Source: BMC Biol. 2022 May 2;20:93. doi: 10.1186/s12915-022-01293-4 (PMC9059368; doi:10.1186/s12915-022-01293-4)
Supplement: Supplementary file 2 — Additional file 2: Table S1. PCR primers. Table S2. The genes with altered expression patterns in the female offspring uteri after prenatal exposure to estriol (E3 v.s CT). Table S3. A The hypomethylated genes in the female offspring uteri after prenatal exposure to estriol (E3 vs CT). B The hypermethylated genes in the female offspring uteri after prenatal exposure to estriol (E3 vs CT). Table S4. The genes with altered E2 responsiveness in the female offspring uteri after prenatal exposure to estriol (E3-E2 vs CT-E2). Table S5. The potential binding proteins with Flag-ERα, Flag-ERβ in Ishikawa cells by Multi-Dimensional Protein Identification Technology (MudPIT). Table S6. The genes that are bound by the ERα-SUZ12 and ERβ-SUZ12 protein complex in Ishikawa cells. The Re-ChIP results show that E3 treatment influences binding of ER-SUZ12 complex to their target genes in Ishikawa cells. Table S7. The genes with altered expression patterns (≥ 1.5 or ≤ -1.5-fold change) in the female offspring cortex and hippocampus after prenatal exposure to estriol (E3 vs CT). Table S8. The top 3000 CpG sites in the female offspring prefrontal cortex after prenatal exposure to estriol (E3 vs CT). Table S9 The genes that are bound by the ERα-DNMT1, ERβ-DNMT1, ERα-SUZ12 and ERβ-SUZ12 protein complex in SY5Y cells. By a Re-ChIP assay, we identified 2391 DNA promotor sites that were bound by both ERα and DNMT1 simultaneously, 53 sites were bound by both ERβ and DNMT1; 253 sites were bound by both ERα and SUZ12, and 260 sites were bound by both ERβ and SUZ12, after E3 treatment. Table S10. A) The top 2000 hypermethylated differential CpG sites in the female offspring hippocampus after prenatal exposure to estriol (E3 vs CT). B) The top 2000 hypomethylated differential CpG sites in the female offspring hippocampus after prenatal exposure to estriol (E3 vs CT). [file 12915_2022_1293_MOESM2_ESM.zip › Additional File 2_ Table S1.docx]

#### Additional File 2: Table 1

#### RT-PCR primers

| Spink 3 | Forward | GACAATGAAGGTGGCTGTCA |
| --- | --- | --- |
|  | Reverse | GGCAACTAGCCTCTTTTCCA |
| Hp | Forward | GTATGTCATGCTGCCTGTGG |
|  | Reverse | GTACCAGGTGTCCTCCTCCA |
| Lcn2 | Forward | CTACAATGTCACCTCCATCC |
|  | Reverse | CCTTGGTTCTTCCATACAGG |
| Cfd | Forward | TGCACAGCTCCGTGTACTTC |
|  | Reverse | CACCTGCACAGAGTCGTCAT |
| Adipoq | Forward | CTCTAAAGATTGTCAGTGGATCTG |
|  | Reverse | ACGTCATCTTCGGCATGACT |
| Prss29 | Forward | GTCAAGCTGCCCTCTGAGTC |
|  | Reverse | TGGTTGCCTGCACATAACAT |
| Trpv6 | Forward | CACAGACAAGAGTCCTGGGC |
|  | Reverse | ACATCGTTTTCTTTGGCAGC |
| Arg1 | Forward | AGAGATTATCGGAGCGCCTT |
|  | Reverse | TTTTTCCAGCAGACCAGCTT |
| Muc1 | Forward | TACCAAGCGTAGCCCCTATG |
|  | Reverse | TGCTCCTACAAGTTGGCAGA |
| Clca3 | Forward | CCAACTGAACAACAACGGC |
|  | Reverse | GCCTGAGTCACCATGTCCTT |
| Grwd1 | Forward | GCAGAAACCTCAGCTGGAAC |
|  | Reverse | CTGACCATACTCCAGCCACA |
| Scx | Forward | CCCAAACAGATCTGCACCTT |
|  | Reverse | CTGTGGACCCTCCTCCTTCT |
| Ssr3 | Forward | CCGGAAGGAGAAAGACGAA |
|  | Reverse | ACCAGAACCAGGAACAGAGTG |
| Dhcr24 | Forward | ACTACTACCACCGACACACGC |
|  | Reverse | CGAAGAGGTAGCGGAAGATG |
| Spsb1 | Forward | CGGTGGCTTTTTCTTCTCAG |
|  | Reverse | ACCCCTAAGGGAGGTTGCTA |
| Hoxc6 | Forward | TCCAGATTTACCCCTGGATG |
|  | Reverse | CAGGGTCTGGTACCGAGAGTA |
| Tnc | Forward | AAAACCATCAGTACCACGGC |
|  | Reverse | AGTCCAGGACAGACGGAAAC |

| Drd2 | Forward | CAGACAGGCCCCACTACAAC |
| --- | --- | --- |
|  | Reverse | TGCTGAATTTCCACTCACCCA |
| Agrn | Forward | GGACTCAGAAGGCTCCAACTGT |
|  | Reverse | GTCAGCCATCTCTGGTGTGTAG |
| Bdnf | Forward | GGCTGACACTTTTGAGCACGTC |
|  | Reverse | CTCCAAAGGCACTTGACTGCTG |
| Tac1 | Forward | TGGCCAGATCTCTCACAAAA |
|  | Reverse | TTTCGTAGTTCTGCATTGCG |
| Cck | Forward | ACTGCTAGCGCGATACATCC |
|  | Reverse | TTATTCTATGGCTGGGGTCC |
| Npy2r | Forward | GAACTGGTTCTGAAGATGGGC |
|  | Reverse | CACTTCTCGGTACAGGCCAC |
| Prkcg | Forward | ATCCGGGCTCATGGCTTTTT |
|  | Reverse | TCAAAGTTTTCGCCACTGCG |
| Dusp1 | Forward | TGCAGCTCCTGTAGTACCCC |
|  | Reverse | CTTCCGAGAAGCGTGATAGG |
| Cart | Forward | CGAGAAGAAGTACGGCCAAG |
|  | Reverse | GGGACAGTCACACAGCTTCC |
| Penk | Forward | ACGCTCTTCCAGTAACCTGC |
|  | Reverse | TGCACGCCAGGAAATTGATG |
| C-Fos | Forward | TCCTACTACCATTCCCCAGC |
|  | Reverse | TGGCACTAGAGACGGACAGA |
| Adora2a | Forward | GCAGAGTTCCATCTTCAGCC |
|  | Reverse | CCTTCATACCCGTCACCAAG |
| Grm2 | Forward | CTGGGAATCAGAGGGGAGAAAT |
|  | Reverse | AACCACGGGACAAACTACTGG |
| Tacr1 | Forward | TCACCTACGCAGTCCACAAC |
|  | Reverse | GGGATGTTCTGGCCACTCAA |
| Fmr1 | Forward | GGACCCCAGAAACCTGAACC |
|  | Reverse | TGGCATTAGCGATGCTGTCT |
| Ppia | Forward | AGCTCTGAGCACTGGAGAGA |
|  | Reverse | GCCAGGACCTGTATGCTTTA |

| Dlgap3 | Forward | CTCAGTATGGGAGTGGGCTG |
| --- | --- | --- |
|  | Reverse | CCCCTTTCTGAAGGAGCCAG |
| Fosb | Forward | CCAGGGTCAACATCCGCTAA |
|  | Reverse | GAAGGGCTAACAACGGGGAA |
| Htr1a | Forward | TGTGAGAGCAGTTGCCACAT |
|  | Reverse | CTTCCTTGAATCGCCGAGGT |
| Adora1 | Forward | GCGACTGCCCAAGGTTTTGA |
|  | Reverse | CTGTCTTGTACCCAAGGTCACAC |
| Bag1 | Forward | AGTGACCCGTAGCAAGAACG |
|  | Reverse | TTCCTCCGTCTGGACCATCT |

**Chip-qPCR Primer**

| AR | Forward | GACCTACCGAGGAGCTTTCC |
| --- | --- | --- |
|  | Reverse | GGGCTAGTCTCTTGCTGCTG |
| Dctn1 | Forward | GCCAAGGACAGGAGAGAAGA |
|  | Reverse | TAAAGCAGCGTCTGAACAGC |
| Agrn | Forward | GCAGTTGCACTGTGTCCAGA |
|  | Reverse | GAACCCACCAGAAGGAAGAA |
| Fos | Forward | TGGCACCCCTTTCTTACCTC |
|  | Reverse | TCCCGACCCTCAGAGAGATT |
| Nr4a2 | Forward | TGGACAGGCAAAAGGGACC |
|  | Reverse | ATAAACAAAGGCACATTGGCGG |
| Timp2 | Forward | AAATTAGCCGGGCGTGGT |
|  | Reverse | TGGCCAGAGACCAACACATA |
| Mcu | Forward | TGTGTGTGTGTGTGAGAGAGAGA |
|  | Reverse | TGGCCAGAGACCAACACATA |
| S1pr2 | Forward | GTCGGGCCCAGTCTTCTATC |
|  | Reverse | GTGTCCATCATTTCCGCAGG |
| Erbin | Forward | CAGTGCCTGCCACATAGAAC |
|  | Reverse | TTCCAAGCAAATAAACTCGAATGGC |
| Ugcg | Forward | GGTTCGTCCTCTTCTTGGTGC |
|  | Reverse | CCTGCGGTCCCTCACTCA |
| Rbfox1 | Forward | GGATATACAGTGCCTGACCCT |
|  | Reverse | ACCTCCAAAGAAGCAGACAGC |
| Impa1 | Forward | GGCTACTCGCAACAGGAAGT |
|  | Reverse | GAGGCAGTATCCCATGCTCC |
| TNK2 | Forward | AGAGACTGACCACATCGAGA |
|  | Reverse | GAATGGCTGGGTCATGTGGT |
| Atxn2 | Forward | GAGGAGACCGAGGACGAGG |
|  | Reverse | CTGCCAATGTCCGCAAGC |
| Adora2a | Forward | TCCCAGATCCTCCATCCGT |
|  | Reverse | GGCCCGTAACTTGGGACAGA |
| Plxna2 | Forward | ATTTCGAGGTTTGCTCCGCT |
|  | Reverse | GAGAGAGACCCCGAGAGCTT |
| Ngfr | Forward | AGCCCCATCAGTCCGCAAAG |
|  | Reverse | CTCCGGCTAACACTCACCCC |
| Fscn1 | Forward | TTTGTGGAGCGCTGCGG |
|  | Reverse | GGTCATGGTGGCAGTAGACG |
| Phgdh | Forward | TGTGCTGCACCCATTAACTC |
|  | Reverse | CCAAACACCGCATATTCTCA |
| Pdef4d | Forward | AGGCACCAACACATTTTAGCAT |
|  | Reverse | TGGGGAAGGGCACACATGAAT |

| Tenm4 | Forward | GCACTGGGTGTATATGAGATGC |
| --- | --- | --- |
|  | Reverse | AGAAGATCATGAAGGAAGTTGTCT |
| Col8a2 | Forward | GGCATGGTGGTGTATACCTG |
|  | Reverse | GTGAGCTGCTGTGCTTAGCC |
| Ggt | Forward | TTGGTCTAATCTGCCTCCCG |
|  | Reverse | ACCTGAACTCTGAAAGCCTCG |
| Syne2 | Forward | TGATGCCATATTGAGTGATTTGG |
|  | Reverse | ACAGCTATTGATTTCCATCTCAGC |
| Ifrd1 | Forward | ATGACTTGGCAGTCCTTTTATTA |
|  | Reverse | CCTGCCTGTGGACAAGAGTA |
| Atoh7 | Forward | TGGGCAACAAAGTATGACCCT |
|  | Reverse | CTAGAGCAGGTTAGAAAAAGTGTCC |
| Lims1 | Forward | GTTTTCCAGGGTGGTAGGGC |
|  | Reverse | TGTCGCTGCATGTCTTACCG |
| Met | Forward | CCATGTAGTTGAGCGGTTTTGA |
|  | Reverse | TGTCAACATTAGACAGATCAACGA |
| Kel | Forward | AGATGAGCGGTCAGAGAGAT |
|  | Reverse | GGCTCAATGGTTAAGGCTTTGG  Fto |
| Fto | Forward | CCAGGCTGGAATGCAGTGG |
|  | Reverse | AAATTGGCAGTGGTGTGTGC |
| Slc1a3 | Forward | TGCCCAGAGCACTTTGCTTAT |
|  | Reverse | AACACAAGTGCCCACAGTACA |
| Olig2 | Forward | ATCTGCGAACCCAAGCAATG |
|  | Reverse | ATCAGGTCCCGGAGAGAACA |
| Nfix | Forward | CCCTGTGTGCATGGCTAGATG |
|  | Reverse | ACACACTGACCAACTGATGCC |
| Tcf12 | Forward | TCTTGAGGGCAGTATGGGTT |
|  | Reverse | TGTGCATTTCTCACATGCCG |
| Hexb | Forward | GTGAGCATTGTGAAGACTGCAT |
|  | Reverse | CAAATTCCCAGTTGCCCCAAG |
| Bdnf | Forward | CCGCTTAAAGGGAACGCCG |
|  | Reverse | GGGAGGAGGTGAGGACAGG |
| Tdp2 | Forward | GGAGAAAATGACCCGTTGGA |
|  | Reverse | ACTTAGAACCTGCCTTTCCAGG |
| Gas7 | Forward | GGAAGCAGAGACTCGTTGGC |
|  | Reverse | GAGCTAGGATCCGCGAGCTG |
| Cntn4 | Forward | CGTGCCAGAAGAAAGGGTATG |
|  | Reverse | ACCATCCTCCATCTTGCTCTCT |
|  |  |  |
