# Supplementary material for: The steroid hormone estriol (E3) regulates epigenetic programming of fetal mouse brain and reproductive tract
Source: BMC Biol. 2022 May 2;20:93. doi: 10.1186/s12915-022-01293-4 (PMC9059368; doi:10.1186/s12915-022-01293-4)
Supplement: Supplementary file 3 — Additional file 3. Uncropped blots. [file 12915_2022_1293_MOESM3_ESM.pdf]

The steroid hormone estriol (E_3) regulates epigenetic programming of fetal mouse brain and reproductive tract

Zhou et al

This file contains uncropped immunoblots

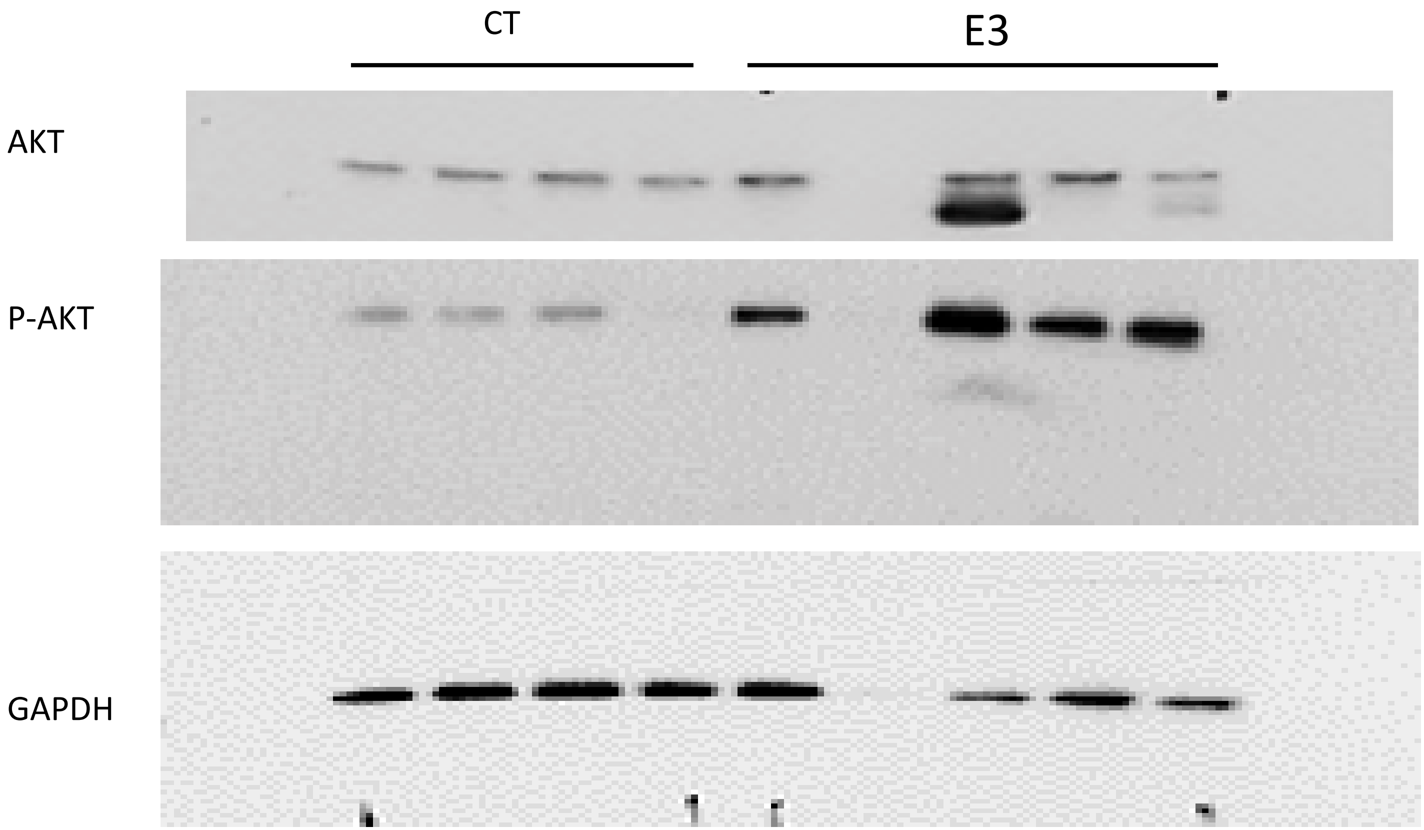

Fig. 2C) Immunoblots of total and phosphorylated (p) Akt in the uteri of vehicle (CT) and E_3-treated mice (E_3).

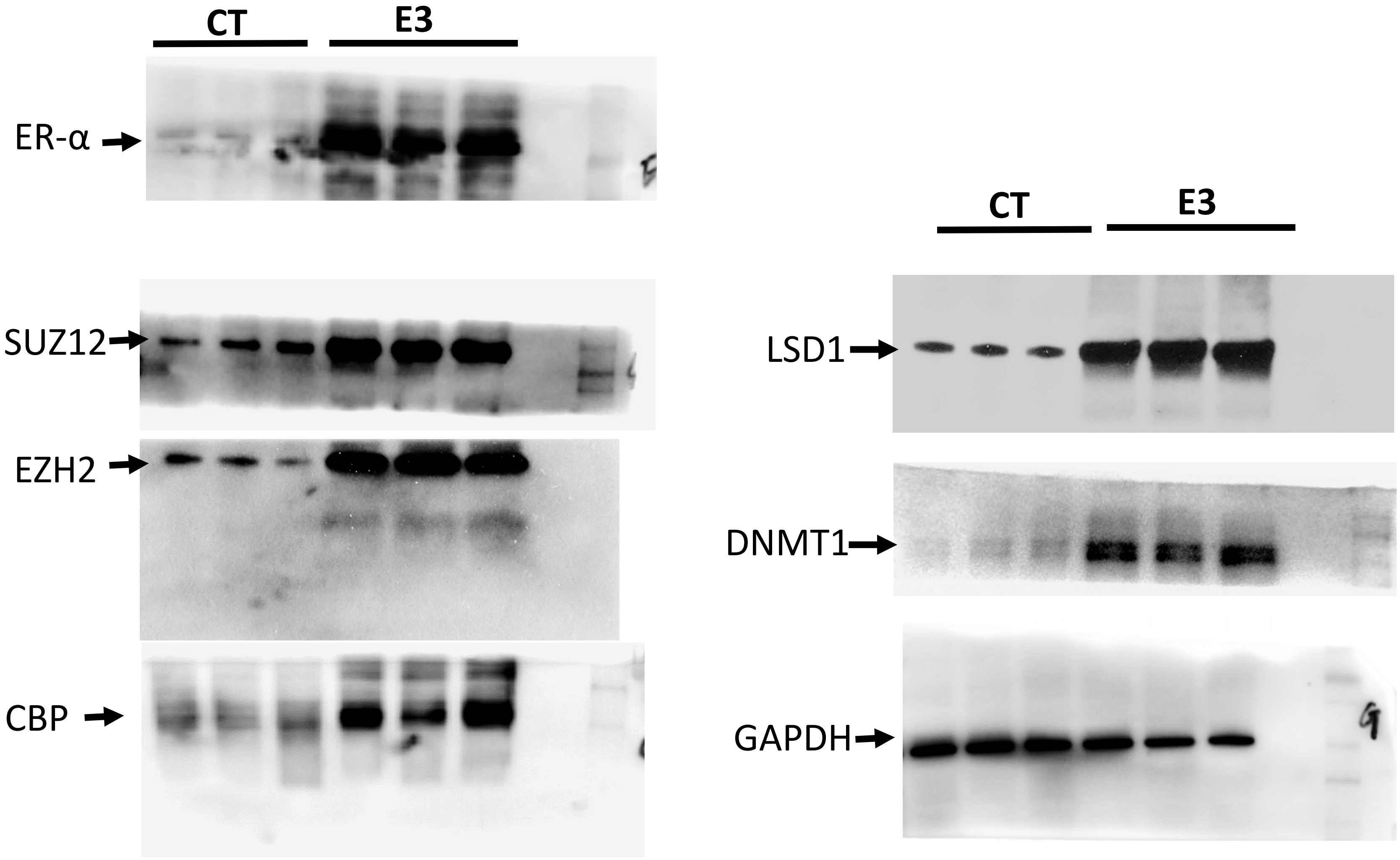

Fig. 3A) Immunoblots of epigenetic modifying proteins from the uteri of prenatally vehicle and E_3-treated mice

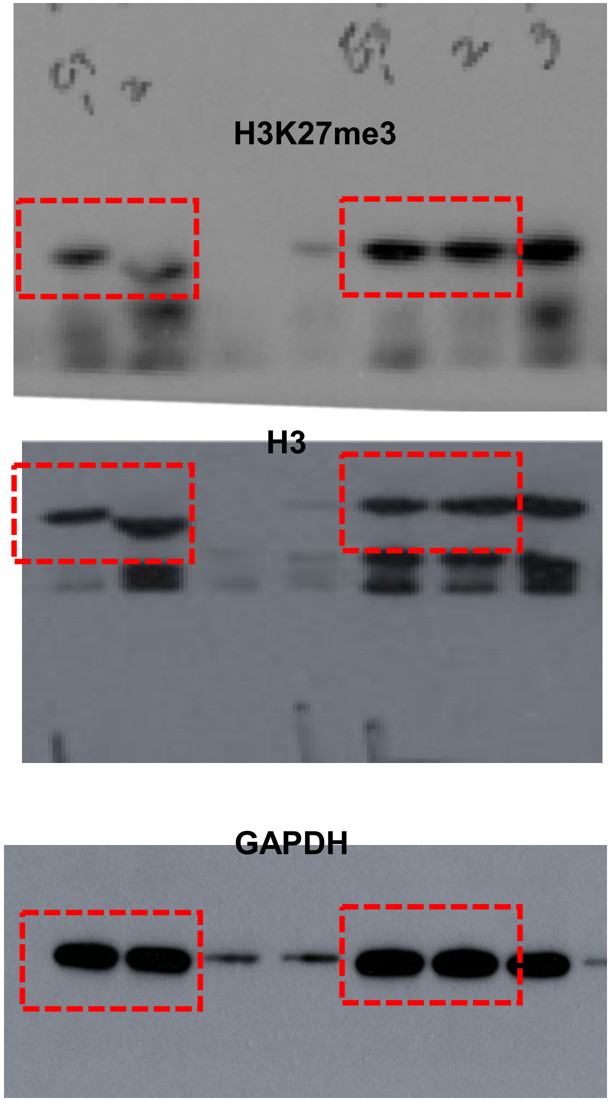

Fig. 3B) Increased histone methylation in prenatally E_3-treated mice (n=2 mice) compared to those treated with vehicle control

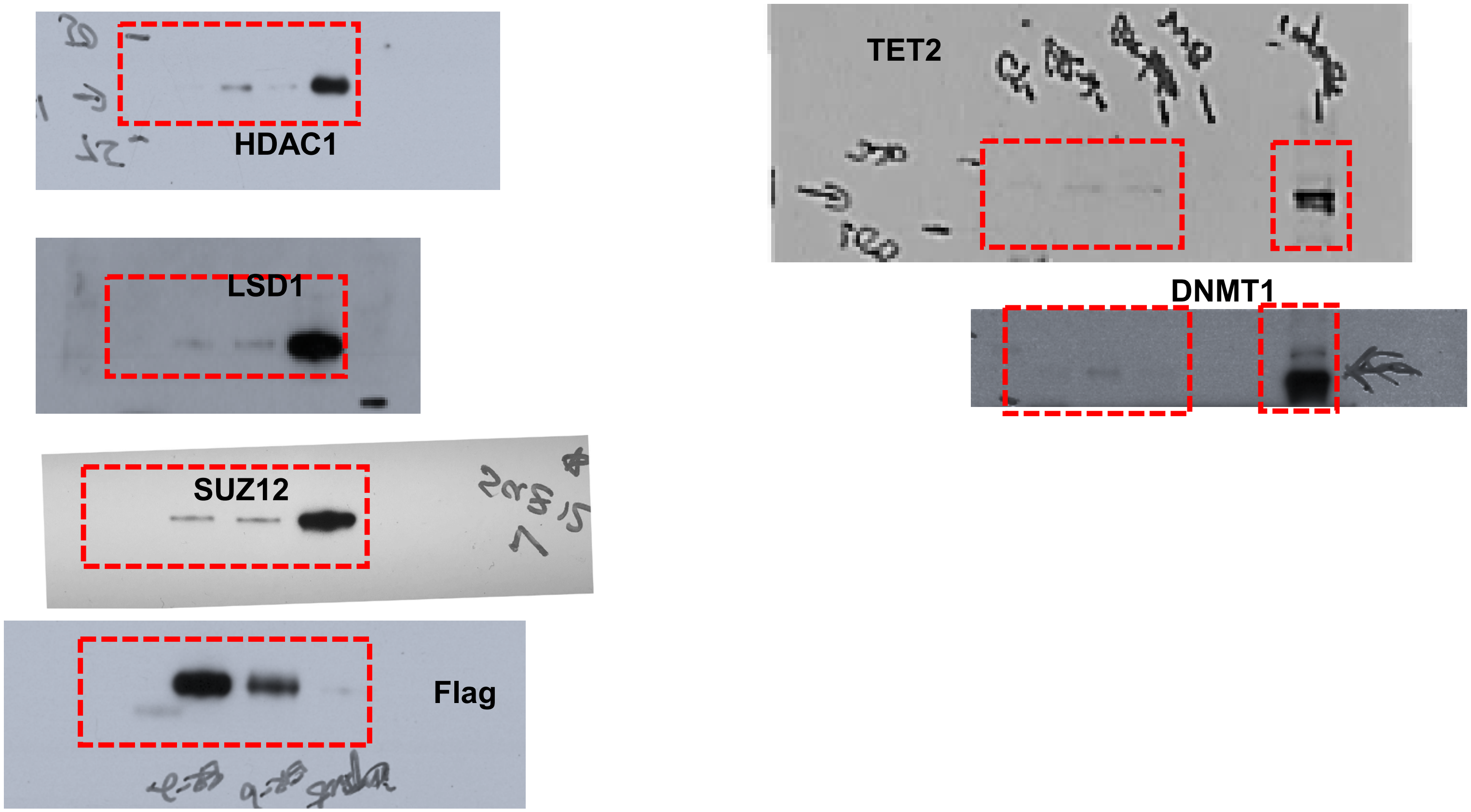

Fig. 3C) Co-immunoprecipitation (IP) of ERα/β with DNA/histone modifiers from Ishkawa cells

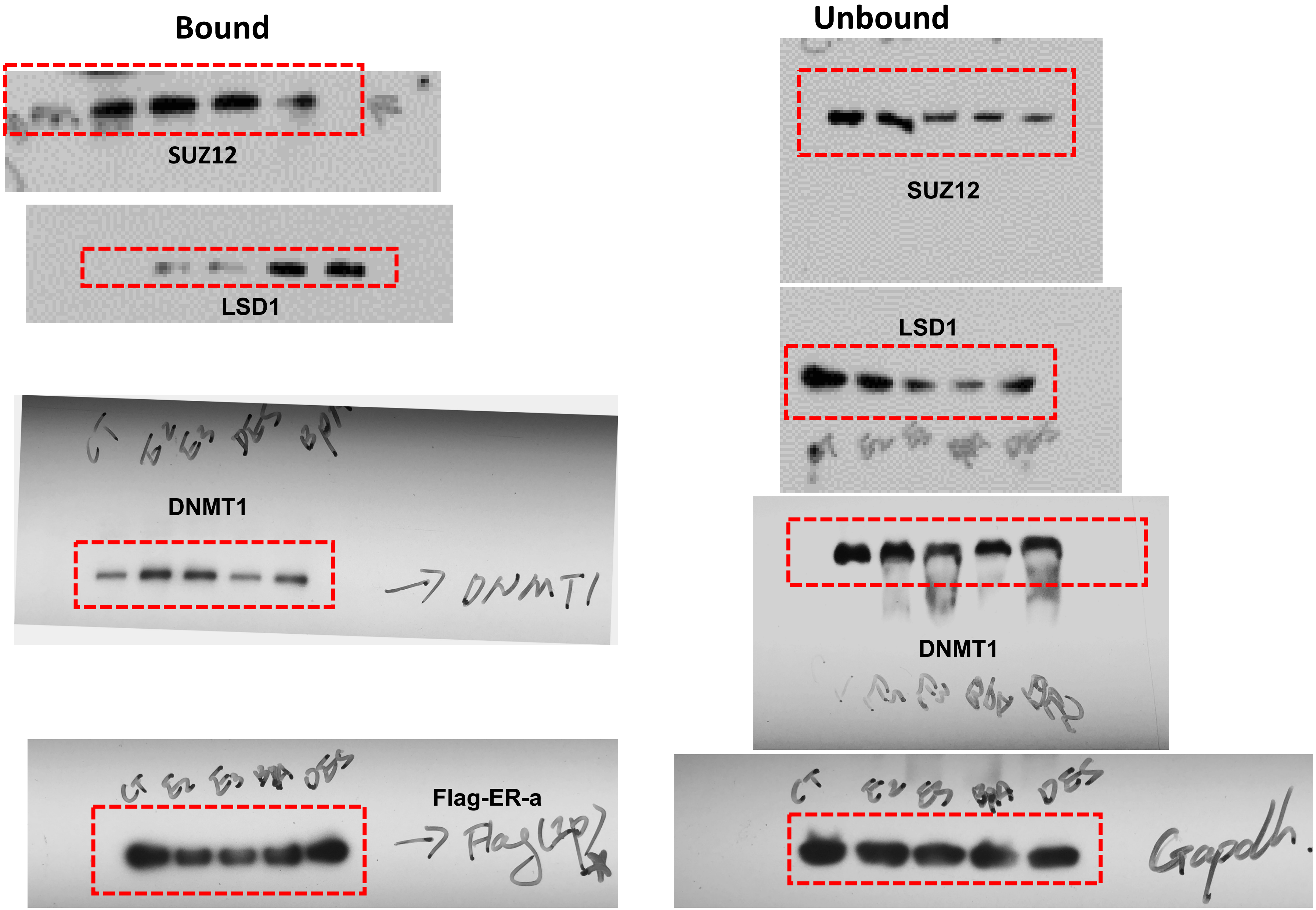

Fig. 3D) Co-IP of Flag-ERα with DNA/histone modifiers from Ishkawa cells treated with CT, E2, E3, BPA or DES

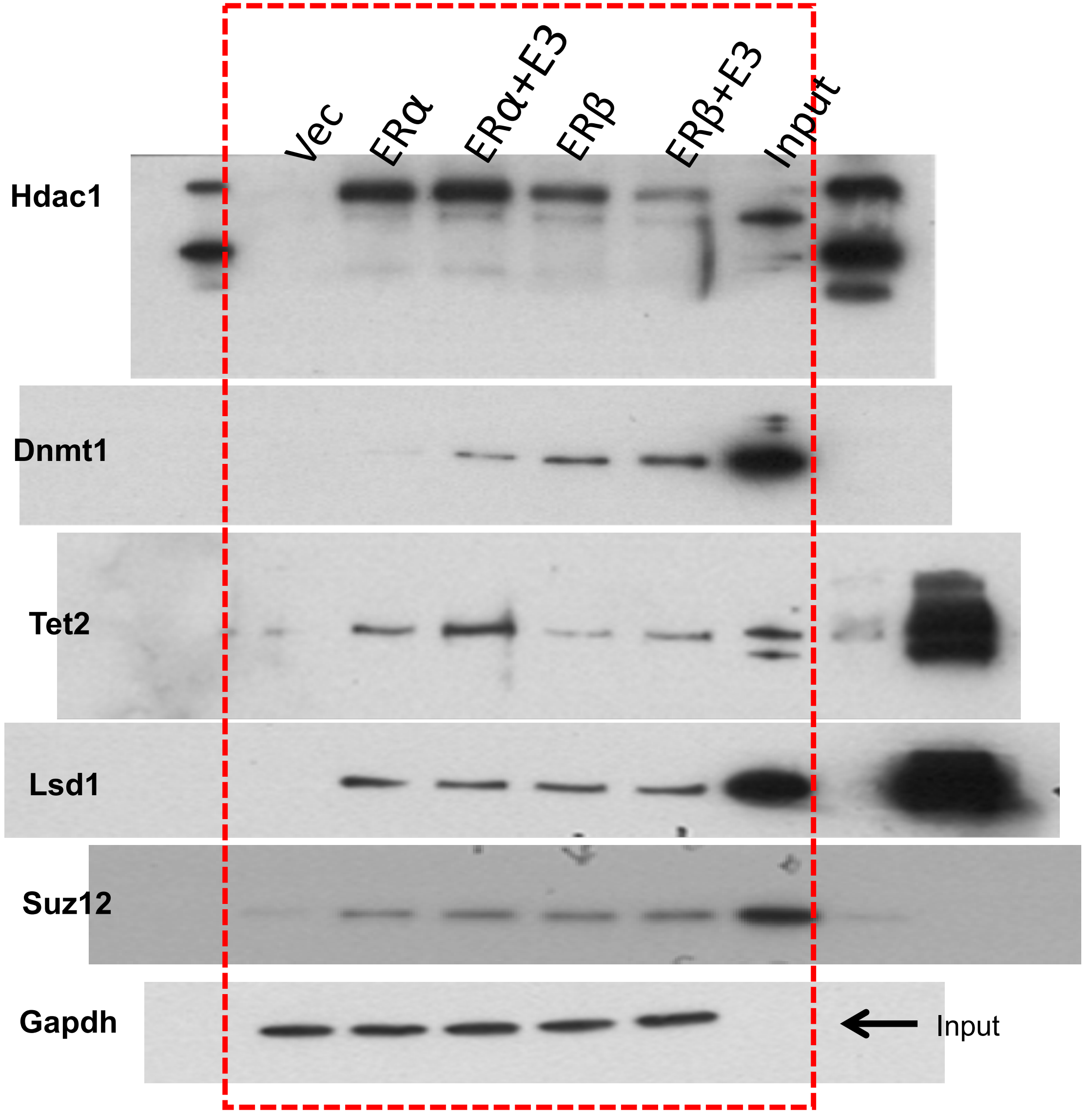

Fig. 8A) Co-immunoprecipitation (IP) of ERα/β and DNA/histone modifiers.
